# Bayesian reassessment of the epigenetic architecture of complex traits

**DOI:** 10.1038/s41467-020-16520-1

**Published:** 2020-06-08

**Authors:** Daniel Trejo Banos, Daniel L. McCartney, Marion Patxot, Lucas Anchieri, Thomas Battram, Colette Christiansen, Ricardo Costeira, Rosie M. Walker, Stewart W. Morris, Archie Campbell, Qian Zhang, David J. Porteous, Allan F. McRae, Naomi R. Wray, Peter M. Visscher, Chris S. Haley, Kathryn L. Evans, Ian J. Deary, Andrew M. McIntosh, Gibran Hemani, Jordana T. Bell, Riccardo E. Marioni, Matthew R. Robinson

**Affiliations:** 1 Department of Computational Biology, University of Lausanne, Lausanne, Switzerland; 2 Centre for Genomic and Experimental Medicine, Institute of Genetics and Molecular Medicine, University of Edinburgh, Edinburgh, UK; 3 MRC Integrative Epidemiology Unit, University of Bristol, Bristol, UK; 4 Population Health Sciences, Bristol Medical School, University of Bristol, Bristol, UK; 5 Department of Twin Research and Genetic Epidemiology, King’s College London, London, UK; 6 Institute for Molecular Bioscience, University of Queensland, Brisbane, QLD Australia; 7 MRC Human Genetics Unit, Institute of Genetics and Molecular Medicine, University of Edinburgh, Edinburgh, UK; 8 Centre for Cognitive Ageing and Cognitive Epidemiology, University of Edinburgh, Edinburgh, UK; 9 Department of Psychology, University of Edinburgh, Edinburgh, UK; 10 Division of Psychiatry, University of Edinburgh, Royal Edinburgh Hospital, Edinburgh, UK; 11 Institute of Science and Technology Austria, Klosterneuburg, Austria

**Keywords:** Statistical methods, Epigenomics, Predictive markers

## Abstract

Linking epigenetic marks to clinical outcomes improves insight into molecular processes, disease prediction, and therapeutic target identification. Here, a statistical approach is presented to infer the epigenetic architecture of complex disease, determine the variation captured by epigenetic effects, and estimate phenotype-epigenetic probe associations jointly. Implicitly adjusting for probe correlations, data structure (cell-count or relatedness), and single-nucleotide polymorphism (SNP) marker effects, improves association estimates and in 9,448 individuals, 75.7% (95% CI 71.70–79.3) of body mass index (BMI) variation and 45.6% (95% CI 37.3–51.9) of cigarette consumption variation was captured by whole blood methylation array data. Pathway-linked probes of blood cholesterol, lipid transport and sterol metabolism for BMI, and xenobiotic stimuli response for smoking, showed >1.5 times larger associations with >95% posterior inclusion probability. Prediction accuracy improved by 28.7% for BMI and 10.2% for smoking over a LASSO model, with age-, and tissue-specificity, implying associations are a phenotypic consequence rather than causal.

## Abstract (web summary)

Linking epigenetic marks to clinical outcomes promises insight into the underlying processes. Here, the authors introduce a statistical approach to estimate associations between a phenotype and all epigenetic probes jointly, and to estimate the proportion of variation captured by epigenetic effects.

## Introduction

Data characterizing gene expression, protein structure, or epigenetic modifications such as DNA methylation, histone marks and nucleosome positioning are becoming increasingly available. Epigenetic marks reflect a wide range of environmental exposures and genetic influences, are critical for regulating gene and non-coding RNA expression^1^, and have been shown to be associated with disease^2^. The identification of clinically relevant epigenetic loci can provide insight into the molecular underpinning of disease^3^, leading to identification of biologically relevant therapeutic targets^4^ and potentially epigenetic-guided clinical decision making^5^.

Most studies testing for association between genomic data and complex traits utilize methodology from genome-wide associations studies, meaning that probe effects are tested one at a time^6^. This methodology does not account for correlations among probes and leads to model over-fitting, poor effect size estimation, and poor calibration of prediction owing to omitted variable bias^7^. In addition, data structure such as intra-sample cellular heterogeneity, sample relatedness, population stratification, or experimental design effects are a major challenge^8^ and result in more cross-chromosome correlation than genetic data. This structure, in conjunction with the fact that cases and controls typically differ in their cell-type composition, can result in spurious associations and many statistical algorithms have been proposed to tackle these potential biases^7,9,10^. However, all current statistical approaches rely upon corrections for structure that require a choice of either a suitable reference profile of representative cell types, or a limited number of pre-selected variables computed from the methylation data (e.g., LFMM2^11^ or ReFACTor^12^), with the underlying assumption that all confounders are reflected by a sparse set of latent covariates and methylation sites.

Here, we present an alternative approach, based on Bayesian inference, that: (i) estimates probe effects on an outcome jointly whilst adjusting for other covariates such as sex and age, avoiding model over-fitting and controlling for both data structure (including cell-count effects) and correlations among probes; (ii) does not require any knowledge of cell-type composition or any selection of proxy confounder variables (i.e., accounts for both known and unknown confounders); (iii) estimates the total proportion of disease risk accounted for by the probe effects (cumulative proportion of variance explained); (iv) estimates probe effects conditional on other sources of data such as single-nucleotide polymorphism data, enabling a determination of the unique contribution of different data; (v) gives an in-depth understanding of the genome-wide range of probe effects in terms of the likely number of independent effects and their variance explained; (vi) can incorporate genomic annotation information into the analysis when estimating probe effects, facilitating unique genome-wide enrichment analyses, describing the variance explained and number of trait-associated probes of each annotation; and (vii) provides improved estimation of biomarker effects, which could be used for disease risk assessment. The approach is similar to, but more flexible than linear mixed model analyses recently proposed in genome-wide association studies^13^, given that we can assign different prior distributions to different sources of variance (individual covariates or groups of covariates). We demonstrate properties (i) through (vii) with theory, simulation and then empirical analysis of body mass index (BMI) and smoking behavior for 9448 individuals with methylation probe measures from whole blood^14^.

## Results

### Methods overview

**Fig. 1:**
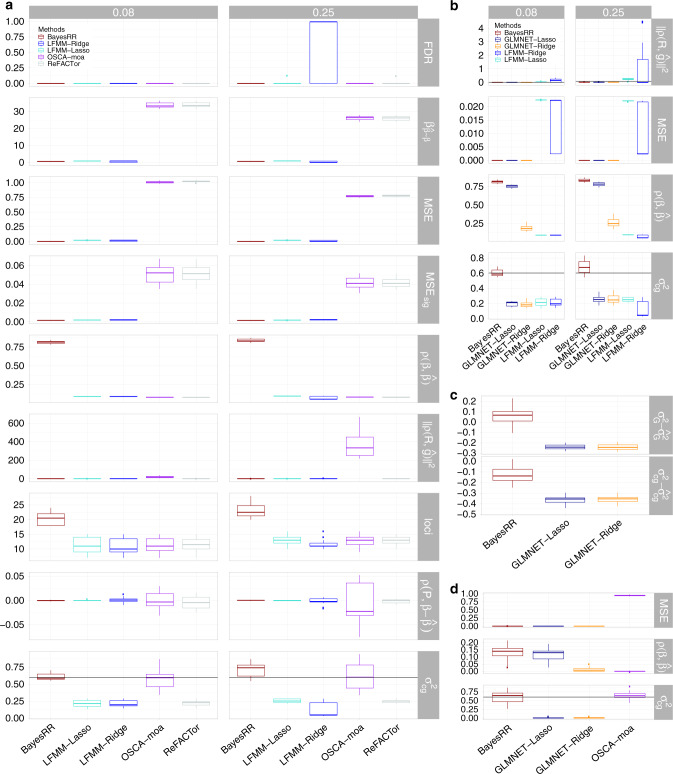
**Simulation study**. Boxplots of distribution of scores, the line in the middle of the box represents the median, upper, and lower bounds of the box represent first and third quartiles respectively, whiskers represent datum up to 1.5 interquartile distance from box bounds. **a** Estimation of phenotype-epigenetic associations using five recent approaches, BayesRR in Brown, to OSCA-moa in magenta, ReFACTor in gray, LFMM-Lasso in Cyan and LFMM-Ridge in Blue; where probes are associated with cell-type proportion variation and the norm of the correlation vector between the phenotype and the cell-type proportions have two different values either 0.08 or 0.25. Row panels provide results for different metrics of performance: the correlation between true effects and estimates ($\rho (\beta ,\hat{\beta })$), the slope of a regression of the estimates on the true effects (${\beta }_{\hat{\beta } \sim \beta }$), the number of genome-wide significant probes identified (loci), the mean square error (MSE), the MSE of the genome-wide significant probes (*MSE*_*sig*_), the false discovery rate (FDR), the norm of the correlation vector between a individual-level predictor made from the probe effects and the cell-type proportions ($| | \rho ({\bf{R}},\hat{{\bf{g}}})| |$), the correlation between the first principal component of the probe data and the difference between the estimated and true effect (∣∣*ρ*(**P**,  **R**)∣∣) and the phenotypic variance attributable to the probes (${\sigma }_{cg}^{2}$). Black lines give the true value across panels. **b** Comparison of BayesRR with just the methods, which fit probes jointly (multi-probe methods) either accounting for latent factors (LFMM-Lasso in Cyan and LFMM-Ridge in blue) or not (GLMNET-Lasso in dark-blue and GLMNET-Ridge in dark-yellow). **c** Simulation results of methylation marker effects for a phenotype influenced by both 100 methylation probes and 1000 SNP markers, showing the difference between the true and the estimated phenotypic variance explained by genetic markers (${\sigma }_{G}^{2}-\hat{{\sigma }_{G}^{2}}$) and epigenetic probes (${\sigma }_{cg}^{2}-\hat{{\sigma }_{cg}^{2}}$). **d** Comparisons of approaches that do not fit latent factors within the model when the underlying epigenetic architecture is less sparse (phenotype is influenced by 1000 probes, rather than only 100).

Our approach assumes that the observed phenotype **y** is reflected by a linear combination of genetic effects (*β*_*G*_) estimated from single-nucleotide polymorphism (SNP) data, epigenetic effects (*β*_*cpg*_) estimated from probes on an array, along with age and sex specific effects (*α*, *γ*), such that:

1 $${\bf{y}}=\alpha {\rm{age}}+\gamma {\rm{sex}}+{{\bf{X}}}_{cpg}{\beta }_{cpg}+{{\bf{X}}}_{G}{\beta }_{G}+\epsilon$$

with the effects *β*_*G*_, *β*_*cpg*_, *α*, and *γ* being estimated in a Bayesian statistical model. Unlike previous approaches that assign a mixture of Gaussian distributions and a discrete spike at zero as a prior for all effects, we assign a new set of mixtures to each group, effectively augmenting the number of hyperparameters proportionally to the number of groups of variables (two in the case of SNPs and methylation probes). This allows for non-identifiable effects to be excluded from the model, whereas the rest are estimated jointly. Adjusting for all different covariates and their effects while estimating each individual probe effect, better alleviates problems related to correlations and structure in the marker data as we show in our simulation study (Fig. 1, also see Methods). Distinguishing between groups of covariates helps us better identify the variance attributable to each group, especially in cases where average effects are bigger in one group (as is likely for methylation and genetic markers). This is because each probe estimate is made after adjusting for the SNP markers and the other “omics” probes in the model. Therefore, our model captures, and thus accounts for, genetic relationships in the data^15^ and importantly for “omics” data, the model also automatically accounts and controls for structural effects (such as cell-count effects, experimental batch effects, or population structure), negating the need to add additional controls for cell counts or principal components within the model.

Our software implementation of this modeling framework, (BayesRR, which is freely available, see “Code availability”) is entirely flexible. Unlike existing methods, any number of data sources can be modeled together, each with separate mixtures, making it applicable to any kind of genetic or epigenetic data. Owing to efficient computational implementation (see Methods), it is also entirely scalable to future data sizes. Furthermore, if only epigenetic probe data are available, estimates of the probe effects would still be obtained jointly, avoiding model over-fitting and controlling for both data structure (including cell-count effects) and correlations among probes (Fig. 1, also see Methods). In addition, other major covariates could be included, for example, including genetic loci of large effect such as HLA in immunodisease or APOE4 variant in Alzheimer disease, or latent factors can still be fit alongside alongside the probe data. We provide an example of how the model can be simply extended to allow probe effects to be estimated accounting for genomic annotation information, providing estimates of genomic enrichment that do not reply on post hoc testing. This flexibility is important as data sets will likely be variable in their structure and the degree to which different “omics” measures are correlated.

### Simulation study

**Fig. 2:**
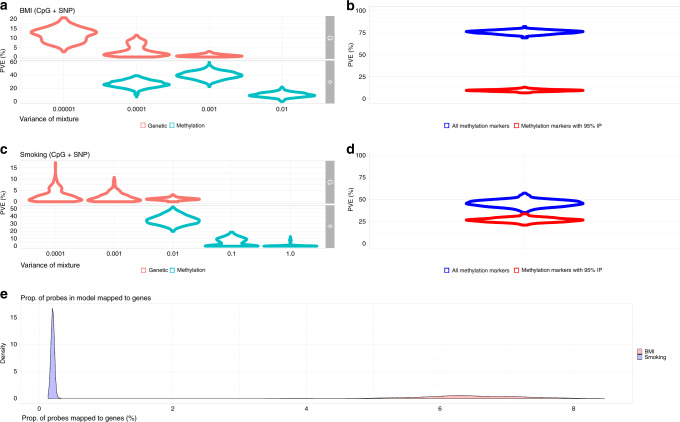
**Biomarker architecture**. Boxplot representing distribution of scores, the line in the middle represents the median, lower bound of the box represents the first quartile, upper bound represents the third quartile, whiskers represent up to 1.5 times the interquartile range from the top or bottom, respectively **a** Phenotypic variance of BMI attributable to the three mixtures for single-nucleotide polymorphism markers (SNP; genetic) and methylation probes, with mixture variances (0.00001, 0.0001, 0.001) and (0.0001, 0.001, 0.01), respectively for BMI, and (0.0001, 0.001, 0.01), and (0.01, 0.1, 1.0) for smoking. **b** For BMI, the phenotypic variance captured by all markers in the model (blue) and for the markers with 95% posterior inclusion probability (IP; red) is shown. **c** For cigarette consumption, the phenotypic variance captured by the mixtures for the SNPs and methylation probes, with same mixture-specific variances as for BMI. **d** Phenotypic variance captured by all markers in the model (blue) and by the markers with 95% IP for cigarette consumption. **e** Distribution of proportion of all methylation probes in model for BMI (red) and smoking (blue).

**Fig. 3:**
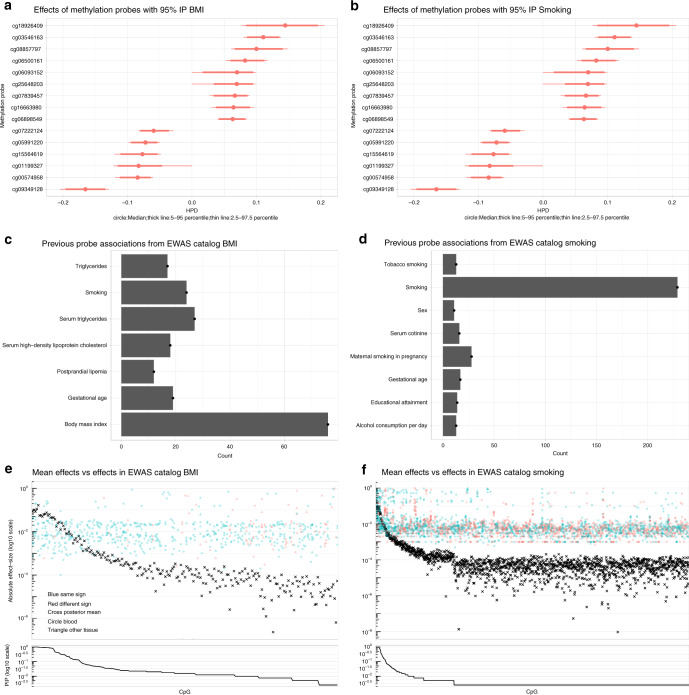
**Annotation replication and enrichment analysis**. **a**, **b** Posterior distribution of effect sizes for methylation probes with 95% posterior inclusion probability (IP) for BMI and smoking, respectively. **c**, **d** Previous associations found for the probes with 95% IP according to the EWAS catalog. **e**, **f** Comparison between mean effect sizes of probes with Posterior Inclusion Probability (PIP)  > 0 and effect sizes from literature in the EWAS catalog. Each cross represents the mean of the posterior estimates, colored point represents an effect size from the EWAS catalog, with blue indicating that the effect size had the same sign as our estimates, in red the contrary case. The shape of the point indicates in which tissue this effect was computed from, circle for Blood and triangle for other tissues. We notice how the agreement between our estimates and literature diminishes as the PIP diminishes, as in our case regularization protects us against overestimation of small effects.

**Fig. 4:**
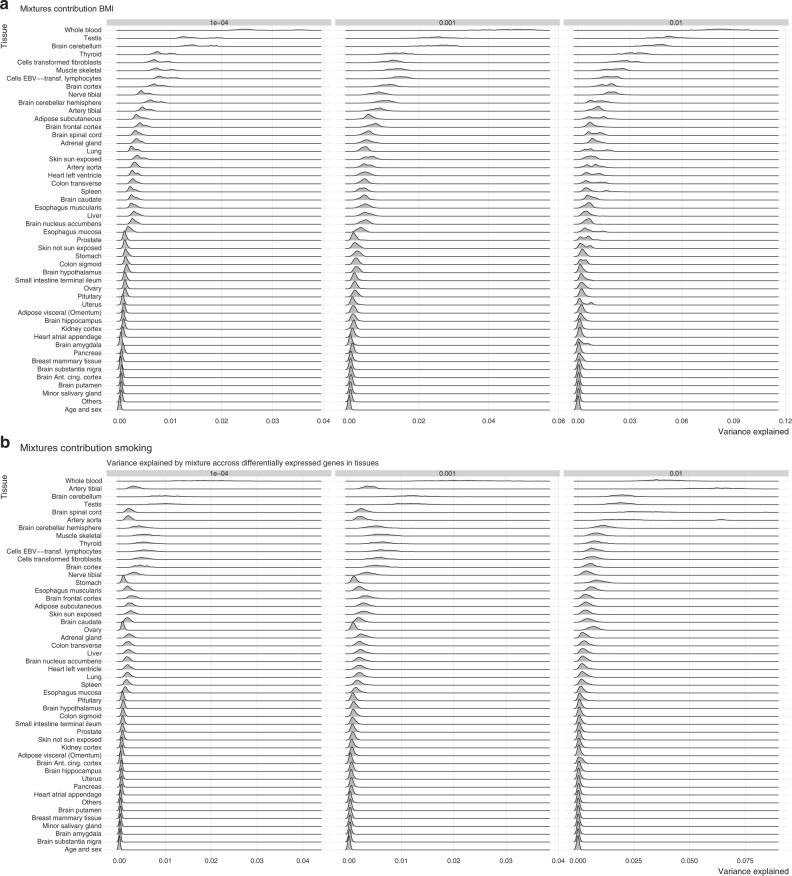
**Variance attributable to tissue-specific gene expression**. **a**, **b** Variance explained by probes that have tissue-specific differentiated gene expression in Genotype-Tissue Expression (GTEx) project. Variance explained is decomposed by the mixture to which the posterior effects belong. For both traits, the biggest contribution comes from probes not mapped into genes differentially expressed in GTEx (not shown). From the probes that mapped into differentially expressed genes, in smoking, the biggest mixture (1% of the total variance explained by probes) captures probes mapping to genes differentially expressed in tibial and aorta arteries, along with the spinal cord, indicating these contribute more than what is observed by the rest of the probes among tissues and mixtures. For BMI, no such difference in effects distribution was observed among mixtures and among tissues.

We simulated methylation data for 2000 individuals at 103,638 probes with five different cell types. We reproduced cell-type proportion variation present in real methylation probe data, using a recently proposed simulation model^12^. Our first simulation scenario, was a sparse setting, where a phenotype is determined by 100 differentially methylated probes (see Methods), which cumulatively explained 60% of the phenotypic variance in the trait. We focus in the main text on two scenarios where probes are associated with cell-type proportion variation and the norm of the correlation vector between the phenotype and the cell-type proportions is either 0.08 or 0.25. These scenarios reflect different degrees of confounding between phenotype and cell-type proportions. We then conduct additional simulations with a wide a range of settings, varying the cell-type proportions, the proportions of differentially methylated probes, the variance of differentially methylated probes, and the variance of the measurement noise and we present these within the Supplementary Information (Figs. 1–4 and see Methods).

We benchmarked our BayesRR approach against four recently proposed methods: single-probe least squares regression, which estimates probe associations one-by-one whilst correcting for sparse latent factors to control for cell proportion confounding (ReFACTor^12^), single-probe mixed linear model association test that estimates probe associations one-by-one conditional on a relationship matrix estimated from the probe data (OSCA-moa^13^), a multi-probe ridge regression that estimates all probe associations jointly and conditionally on latent factors (LFMM2-ridge^11^), and a multimarker LASSO model, which estimates all probe associations jointly and conditionally on latent factors (LFMM2-lasso^11^). Our BayesRR approach outperforms these approaches as it estimates phenotype-probe associations more accurately with higher correlation of the estimated effects with the true simulated values and with lower mean square error (MSE, Fig. 1a). This results in almost twice the number of methylome-wide significant discoveries at  >95% posterior inclusion probability (IP) within this data, whilst controlling for cell-type proportion confounding and maintaining a false discovery rate of much <5% (Fig. 1a). BayesRR controls for cell proportion confounding without a requirement for the addition of latent factors within the model, as evidenced by: (i) the accurate effect size estimates with reduced MSE; (ii) no inflation of the norm of the correlation vector between a individual-level predictor made from the probe effects and the cell-type proportions, and (iii) no correlation between the first principal component of the probe data and the difference between the estimated and true effect, despite significant cell-type proportion confounding within the simulated data (Fig. 1a).

This is further evidenced by comparing LASSO and ridge regression with latent factors implemented in LFMM^11^, to LASSO and ridge regression without latent factors as implemented in glmnet^16^, where we find that that ability to recover the true effects is increased, phenotype-probe associations are better estimated, and cell-type confounding is controlled by the models that do not fit latent factors (Fig. 1b). This is because there are probes in this setting that both influence the phenotype and are associated with cell-type proportions, and thus by removing variation associated with leading latent factors of the data, capacity to detect these probes and estimate their effects accurately is reduced. In this setting, approaches that estimate phenotype-probe association one-by-one such as ReFACTor and OSCA-moa, do not control for correlations in probe effects across the genome, resulting in increased MSE and erroneous correlations between probe effect estimates and cell-type proportion confounding (Fig. 1a). Having shown that multi-probe methods remove the necessity for latent factor correction and that BayesRR performs better than other multi-probe approaches (Fig. 1a, b), we then conduct our remaining benchmarking of BayesRR against LASSO and ridge regression without latent factor correction as implemented in glmnet, finding the exact same increased performance of BayesRR irrespective of the variance of the cell-type proportions, the proportions of differentially methylated probes, the variance of differentially methylated probes, and the variance of the measurement noise (see Supplementary Figs. 1–4).

BayesRR also provides accurate estimation of the total proportion of phenotypic variance explained by the probes, represented by the panel ${\sigma }_{cg}^{2}$ in Fig. 1a across different scenarios. With the exception of a mixed linear model as implemented in restricted effects maximum likelihood within OSCA^13^ (OSCA-moa), all other approaches only enable estimation of the proportion of variance attributable probes identified as genome-wide significant and thus do not provide an estimate of the total association between the phenotype and the probe data (Fig. 1a). We examined whether methylation probe effects can be estimated conditionally on the SNP marker effects to determine the unique contribution of each type of marker. We simulated correlated genetic and epigenetic effects, with 100 epigenetic effects drawn from a normal distribution ${\mathcal{N}}(0,0.5/100)$, and a combination of 100 genetic effects drawn from a normal distribution ${\mathcal{N}}(0,0.2/100)$ and 900 smaller genetic effects drawn from ${\mathcal{N}}(0,0.01/900)$ from a total of 103,638 simulated SNP markers. We find that BayesRR can better distinguish between the variance explained by genetic markers and methylation probes, as compared with LASSO or ridge regression implemented in glmnet, but with higher variability in the error of estimates as compared with when only estimating phenotypic variance associated with methylation probe (Fig. 1c and Supplementary Fig. 4).

We then compared BayesRR with other multi-probe approaches that do not fit latent factors within the model across two levels of sparsity, the first where a phenotype is influenced by 100 probes (Fig. 1a) and the second where a phenotype is influenced by 1000 probes (Fig. 1d). Both the mixed linear model and BayesRR provide unbiased estimates of the proportion of phenotypic variance captured by the probes, with the error variance of each approach dependent upon the underlying effect size distribution (Fig. 1a, d). Again however, estimated effects from the other multi-probe approaches showed reduced correlation with the true effects and higher MSE as compared with BayesRR, demonstrating that BayesRR will provide improved performance in both sparse and non-sparse regimes (Fig. 1d).

### Application to BMI and smoking

We then applied BayesRR to two lifestyle factors, smoking and BMI, that are correlated with numerous health outcomes across the lifecourse. Previous studies have shown that smoking produces a strong alteration in methylation levels, which are related to the etiology of smoking-related disease^17^. BMI has also been associated with methylation levels and adipose-related traits^18^. Here, we present results from a converged set of four models for each trait, each model having different starting values, applied to 9448 individuals of the Generation Scotland cohort.

For BMI, 75.7% (95% CI 71.70–79.3) of the phenotypic variance was captured by methylation probes, with 39.5% (95% CI 28.3–49.7) of this attributable to 509.3 (95% CI 348–663) probes that each explain ~0.1% of the phenotypic variance (Fig. 2, Supplementary Tables 1 and 2). The remaining phenotypic variance captured by methylation probes, was attributable to 10 probes with 95% IP, which cumulatively explain 9.7% (95% CI 7.5–11.9) of the phenotypic variance of BMI (Fig. 2b). This suggests that most epigenetic probe effects for BMI are relatively small, but larger than SNP marker effects, which cumulatively capture an additional 15.8% (CI 11.2–20.5) of the phenotypic variance (Fig. 2a). In total, the variance captured by both methylation probes and SNP markers was estimated as 91.5% (95% CI 87.3–95). For smoking behavior, defined as the number of pack years, we find that 45.6% (95% CI 37.3–51.9) of phenotypic variance is captured by methylation probes (Fig. 2). In contrast to BMI, we find evidence for 17 probes with 95% IP, which capture (26.7%, 95% CI 22.3–30.9) the variance explained by methylation probes (Fig. 2). We observe that 35.15% (95% CI 24–46) of phenotypic variance was attributable to 111.34 (95% CI 87–140) methylation probes of effect size  <1%, whereas there are 3.42 (95% CI 0–9) probes with effect size  <10% explaining 5.5% (95% CI 0–14.27) of the variance (Fig. 2). Of the probes mapped to genes, 6.43% (95% CI 4.91–7.95) are in the model for BMI, >0.2% (95% CI 0.15–0.24) for smoking behavior (Fig. 2). In total, the variance captured by both methylation probes and SNP markers was estimated as 51.5% (95% CI 42.1–58.8). Taken together, these results highlight the ability of our approach to describe the architecture of epigenetic associations, in terms of the likely number and effect size of associated probes, and our results imply a large effective number of epigenetic probe associations, spread throughout the genome, for BMI as opposed to a limited number for smoking behavior.

We performed our analysis both with and without adjustment for the first 20 principal components of the genetic data, the first 20 principal components of the methylation levels and the cell composition, finding practically identical estimates with and without these adjustments (see Methods). We repeated the analysis but excluded close relatives and modeled only the methylation probe effects in 2,614 unrelated individuals, finding 69.55% (95% CI 57.35–78.32) of the phenotypic variance for BMI and 73.83% (95% CI 54.33–88.26) for smoking, respectively. We also used a linear mixed effects model where probe values are used to calculate a co-variance matrix, which is then used in a restricted maximum likelihood estimation (REML) approach^13^, but this approach did not produce a converged set of estimates for either phenotype, with or without relatives in this data. These results imply that our estimates of the variance captured by methylation probes, are independent of the variance attributable to SNP markers, independent of family effects, and independent of data structure captured by the leading principal components of the data.

We then proceeded to derive annotation information from the posterior distribution over effects to provide some biological inference. First, we looked for Gene Ontology (GO) enrichment for the probes whose IP was  >95% (see Methods). From the 20 top-enriched terms, we find those corresponding to thrombin-related pathways and cerebral cortex development for smoking (Supplementary Table 6). From the 20 top-enriched terms, we find those related to ESCRT-II complex, glycoprotein transport, and cholesterol for BMI (Supplementary Table 7).

We looked for the probes with 95% IP, shown in Fig. 3a, b, in the EWAS catalog. We count the appearance of traits in previous associations for each of these probes with 95% IP, the resulting histograms are shown in Fig. 3c, d. We also found associations with triglycerides, cholesterol, and smoking for the probes found in the BMI trait. We found previous associations with Alcohol consumption and educational attainment for the probes found in the smoking trait. From the probes with 95% IP in BMI, all had been previously associated with BMI in the EWAS catalog, for smoking, three probes with 95% IP had not been previously associated with smoking-related traits in the EWAS catalog, these probes are cg00884093, cg0440053, and cg23288337. We further looked in genes associated with these probes, we found gene CELSR1 for which has been associated to chronic obstructive pulmonary disease disk among women^19^; the other gene, ETV5 is member of the oncogenic subfamily of ETS transcription factors^20^; the final gene, ECEL1P2 has been associated with differential methylation levels in smokers even after quitting and lung cancer risk^21,22^. Thus, probes with 95% IP show congruent with previous results along with suggesting two genes whose DNA methylation levels are not directly related to smoking in literature.

We performed a comparison of the magnitude and sign over all mean effect sizes estimated in this study and those in the EWAS catalog over different tissues. The resulting plots Fig. 3e for BMI and Fig. 3f for smoking show in the upper panel the logarithm base 10 of our mean effect estimates as black crosses, with blue points representing previous effect sizes with the same sign as our estimates, and with shape corresponding to the tissue these effects were calculated for, red points indicate that the previous estimate had the opposite sign than our estimates. On the lower panel of both subfigures we observe the PIP of the corresponding probe in the upper panel, we observe how the congruency between our estimates and the EWAS catalog estimates diminishes as the PIP reduces. For our model, the reduction of power to identify the effect is reflected. However, without regularization we run the risk of systematically over-estimating the effect sizes if non-significant effect sizes are unreported, as seems to be the case here. By regularizing, we are better equipped to resolve the estimates in these low power regimes, yielding improved estimation and more efficient use of the data.

We further take advantage of the fact that if we use the posterior distribution over all effects, we can derive a posterior distribution over GO terms and devise a definition of enrichment (see Methods). Under our enrichment statistic, we can measure those GO terms, which explain a greater proportion of phenotypic variance than expected, given the proportion of probes that map to the GO term (Supplementary Fig. 17 for BMI, Supplementary Fig. 18 for smoking). Then, using a ROPE decision rule^23^, we can define a term as being enriched if the IP of the GO term in the model is  >95%, and if 95% of the posterior distribution of enrichment is outside the interval $\left(0.5,1.5\right)$. We sorted significantly enriched terms by their mean enrichment and generated a tree map of the terms using REVIGO^24^. For BMI, there is a preponderance of lipid transport, cholesterol transport, morphogenesis, and above all, regulation of epidermal growth factor-activated receptor activity (Supplementary Fig. 15). For smoking, response to xenobiotic stimulus was enriched (Supplementary Fig. 16). Taken together, this demonstrates the novel findings and additional inference that can be obtained from conducting whole-genome enrichment analyses, rather than testing for enrichment at only those effects that are singularly found to be above a significance threshold.

Third, we took this one-step further by extending our model to group probes according to prior biological information and then estimate the probe effects incorporating genomic annotation. We based our annotations on results by^25^ that computed *t* scores for the specificity of gene expression in every tissue from GTEx consortium^26^. We considered that a gene was differentially expressed (positively or negatively) specifically to a given tissue if the absolute value of its *t* score for that tissue was in the 0.001 or 0.999 quantiles of the *t* distribution. We then mapped the probes to genes and genes to tissues, probes that did not mapped to a gene in the GTEx data were put in a different group. In order to achieve non overlapping associations of probes to tissues (as required for our model), we assigned each gene to the tissue they were the most specific to (i.e., the tissue that had the highest absolute *t* score for that gene). The probes were therefore put in 46 different groups (one for each tissue available in GTEx) when feasible. Some tissues had too few probes associated to them, we therefore lumped them together in group 47 (others) so that every group contained at least 200 probes. Probes that were mapped to genes that do not appear in the array were put in group 48, age and sex were assigned to group 49, and SNPs to group 50. Each one of these groups were assigned the prior mixture variances of (0.0001, 0.001, 0.01). We estimated the variance attributable to each group of probes and further decomposed these estimates by the mixture they belonged (Fig. 4). For both traits, methylation probes of genes differentially expressed in whole blood showed highest variance explained which is expected given the tissue used to generate the methylation data (Fig. 4). Conditional on this, our model partitions the variation attributable to other annotation groups, and across mixture groups within each annotation. For BMI, methylation of genes that are differentially expressed in the adrenal gland, subcutaneous adipose, thyroid, fibroblasts also showed larger effect sizes (Fig. 4). For smoking, we find that probes mapping to genes that are differentially expressed in the aorta, the tibial artery, the brain spinal cord, and lymphocytes are also those with larger probe effects (Fig. 4). Associations for BMI are spread among tissues and among mixtures more so than for smoking (Fig. 4), highlighting the large effective number of BMI associations in blood spread across biological processes.

Finally, we use the estimated methylation probe effects to predict BMI and smoking behavior in the Lothian Birth Cohort 1936 (LBC), the Accessible Resource of Integrated Epigenomics Studies (ARIES) data set and the UK Adult Twin (TwinsUK) Registry (see Methods). We compared the prediction accuracy gained from our approach to recently obtained estimates from another model based on the LASSO estimator, using the *R*^2^ metric. For BMI, we achieve an adjusted *R*^2^ of 19.5% with a slope of 0.72 (0.052 SE) for adult BMI in LBC and 30.83% with a slope of 0.86 (0.041 SE) in TwinsUK (Table 1), reflecting the fact that the age structure of TwinsUK more closely reflects that of GS, then the elderly individuals of LBC. In the ARIES data set, we achieve *R*^2^ of 3.34% with a slope of 0.38 (0.061 SE) for birth weight, 2.05% with slope of 0.3 (0.069 SE) for BMI at age 7, 9.65% with a slope of 0.6 (0.071 SE) for BMI age 15 and up, 24.43% with a slope of 0.84 (0.069 SE) for BMI in adult males, and 18.36% with a slope of 0.7 (0.069 SE) for BMI in adult females (Table 1). Differences in slope (and prediction accuracy) imply differences in the methylation associations across ages and cohorts for BMI, suggesting that methylation associations are a consequence of BMI and are to some degree specific to the lifestyle/diet of individuals at a particular time and place. Overall, these values amount to an improvement of 28.7% in comparison to the LASSO predictor in the LBC. For smoking, we observed an adjusted *R*^2^ of 47.9% with slope of 1.02 (0.058 SE) for LBC and 38.49% with slope of 1.01 (0.057 SE) for males in ARIES, 13.7% more than the LASSO predictor (Table 1). This replicates previous results showing that methylation profiles predict BMI independently of genetic profiles in an additive manner^27^ and shows that our approach can better capture the overall distribution of effects, including small effects, whereas accurately estimating larger effects, leading to improved phenotypic prediction.

**Table 1:** *R*^2^(%) in replication study.

Trait	Method	LBC1936	TwinsUK^a^	A.0	A.7	A.15	A.FOF	A.FOM.
BMI^b^	LASSO	18.7	–	1.07	0.13	2.95	9.37	14.95
	Bayes	19.5	30.83/9.33	3.34	2.05	9.65	18.36	24.45
Smoking^c^	LASSO	42.18^d^	–	–	–	–	24.71	–
	Bayes	47.9	–	–	–	–	38.49	–

A phenotypic predictor was created in the Lothian Birth Cohort data 1936 (LBC1936) and Accessible Resource of Integrated Epigenomics Studies (ARIES) cohorts from the methylation effects estimated in the Generation Scotland (GS) data. The prediction accuracy as measured by the *R*^2^ statistic is presented as compared with LASSO estimates of the methylation effects. In the ARIES cohort, A.0 refers to measures at birth, A.7 refers to measures at age 7, A.15 refers to measures at age 15, A.FOF refers to adult males (fathers), and A.FOM refers to females (mothers).

^a^Whole blood/adipose tissue.

^b^log(kg/m^2^).

^c^log(p.p.y.+1).

^d^Estimates from GS wave 1, *n* = 5000.

We further assessed the variation captured by our predictors in adipose tissue within the TwinsUK data, finding 9.33% *R*^2^ with a slope of 0.4 (0.065 SE, Table 1), implying that methylation associations in whole blood and adipose tissue overlap, but with a degree of tissue-specificity. We also assessed the variation captured by our predictors in other traits finding in LBC wave one that our smoking predictor captures 7.2% of the variance for forced expiratory volume, and our BMI predictor captures 11.2% of the variance of triglyceride levels in blood and 7.6% of the variance of high density lipoprotein levels in blood. These results, along with our enrichment analyses, show that our approach captures a signal related to the relevant biological processes underlying these phenotypes, but that trait-methylation associations show age- and tissue-specificity.

It is important to note that the sample size and variance captured by all of the probe effects in the training data govern the prediction accuracy obtaining in the testing data. Following^28^, the squared correlation between a phenotype in an independent sample and a predictor of the phenotype can be approximated given the sample size of the initial study, the expected variance explained by the covariates, and the effective number of independent covariates. Assuming an effective number of covariates of 20,000 (approximate number of protein coding genes) an *R*^2^ of ~25% is expected for BMI within our prediction samples, which is in-line with the values we obtain here. If the initial study sample size increased to 100,000 individuals than an *R*^2^ of over 60% is expected, which in combination with SNP array data, would lead to a predictor of BMI with an *R*^2^ of ~80% from a single blood test. However, the theory described above does not account for the fact that probe variation is likely a consequence of the phenotype and thus in a regression equation, phenotypic variance will appear on both sides. If the consequential effects are large and there are considerable changes with age that occur^29^, or differences in effects across tissues, then the prediction accuracy obtained from methylation probe data will likely differ across cohorts as demonstrated here.

## Discussion

We present BayesRR, a statistical model for joint inference of genetic and epigenetic effects over complex phenotypic traits. Using simulation, we show that BayesRR outperforms other approaches as it has the advantage of controlling for all factors at once and performing statistical inference jointly on all of the model parameters and adjusting estimates conditionally on each other. By working in a Bayesian framework, we derive a rich representation of the estimated effects through probability distributions, where all markers are taken into account and for which we can assess genome-wide enrichment of relevant biological features. From these distributions, we conclude that from the same set of probes in the same individuals, two example phenotypes show different architecture in the distribution of their effects, with the distribution of effects for cigarette consumption being more concentrated in a few epigenetic markers (15 markers with 95% IP explaining ~26.78%), whereas for BMI, we have more probes associated with the phenotype (17 markers with 95% IP explain only 9.70%). Our genome-wide enrichment analyses, identified blood cholesterol, lipid transport, and sterol metabolism pathways for BMI, and response to xenobiotic stimulus for smoking, all with  >95% posterior IP of having methylation probes with effects sizes  >1.5 times larger than the average. For both BMI and cigarette consumption, a large amount of phenotypic variance is captured by epigenetic markers in the training data set, which may be expected as trait-associated DNA methylation probe variation is likely to a large degree to be a consequence of the phenotype, as evidenced in our enrichment analyses and the prediction results from the ARIES study. These consequential effects lead to the expectation that if applied to common complex disease, the model we present may enable accurate characterization of disease progression and better identification of individuals who are on a path to disease where future diagnosis is likely (i.e., those that are pre-diabetic, in the early stages of dementia, etc.). It remains to be seen whether such large amounts of phenotypic variance can be captured by a methylation array for common complex disease, but our prediction results shows that our approach can better describe the overall distribution of associations leading to improved phenotypic prediction.

There are a number of important considerations and caveats. It is important to punctuate that the inferred associations only relate to the present state of the biomarkers and are not intended to capture any causality between methylation status and outcome. Given the highly variable nature of “omics” measures, the variation across data sets in the degree of confounding by experimental biases or unwanted biological variation that will contribute to the variation captured by probes, and the considerable changes with age that occur^29^, it is highly unlikely that the phenotypic variance attributable to probes is stable across cohorts and with age. Determining biomarkers for future disease outcomes requires a different experimental design, for example, longitudinal studies with a baseline, along with methodological extensions for causal inference within this framework, which our future work will focus on. In addition, while we extend the model to ask how much additional phenotypic variance of each trait can be captured by methylation probes from whole blood above that captured by a SNP array, partitioning the phenotypic variance explained exactly may be difficult in data sets where factors are highly correlated. Furthermore, although we present a whole-genome enrichment approach, identifying novel pathways is currently limited and technological improvements are required to improve our ability to capture, define, and understand epigenetic marker variation. Finally, Bayesian inference comes at increased computational cost and requires the specification of prior distributions, for example here, that effects can be well described by a series of Gaussian distributions. A Student-t likelihood could be a path worth exploring as its inferences could be more robust to outliers^30^ and additionally, although a Gaussian model may still be applied to categorical disease-or-not measurements, developing an extension to model binary response variables and explore performance in unbalanced case–control settings will likely be worthwhile.

In conclusion, our model can be applied to any kind of genomics data providing unbiased estimates of marker effects, conditional on other markers, covariates and on the data structure, without the need for specific cell-type proportion control. By operating in a Bayesian framework, the uncertainties over the estimates given the data are represented explicitly, helping the researcher to interpret and draw conclusions over the architecture of the variance in the trait. We provide freely available software with source code available to facilitate further replication and potential applications of the methodology (see “Code Availability”).

## Methods

### Statistical model

We assume additive probe effects ${\beta }_{cpg}\in {{\mathbb{R}}}^{{{\rm{M}}}_{{\rm{cpg}}}{\!}\times {\!}1}$, genetic effects ${\beta }_{G}\in {{\mathbb{R}}}^{{{\rm{M}}}_{{\rm{G}}}{\!}\times{\!} 1}$, age and sex effects *α*, *γ* associated over a vector of measurements over a trait ${\bf{y}}\in {{\mathbb{R}}}^{{\rm{N}}\times 1}$ such that,

2 $${\bf{y}}=\alpha {\rm{age}}+\gamma {\rm{sex}}+{{\bf{X}}}_{cpg}{\beta }_{cpg}+{{\bf{X}}}_{G}{\beta }_{G}+\epsilon$$

where $\epsilon \sim {\mathcal{N}}\left(0,{\sigma }_{\epsilon }^{2}{{\bf{I}}}_{N}\right)$, the methylation matrix **X**_*cpg*_ and the genotype matrix **X**_*G*_ have been centered and scaled to unit variance. We assume that only a subset of $\Theta =\left\{{\beta }_{M},{\beta }_{G},\alpha ,\gamma \right\}$ have an identifiable effect over trait **y**, as such, and proceeding in a Bayesian framework, we assign a sparsity inducing prior over Θ. The chosen prior follows the formulation of ref. ^31^, which is a mixture of *L* Gaussian probability densities and a discrete “spike” at zero. As such, each Θ_*i*_ ∈ Θ is distributed according to:

3 $${\Theta }_{i} \sim {\pi }_{0}{\delta }_{0}+{\pi }_{1}{\mathcal{N}}\left(0,{\sigma }_{1}^{2}\right)+{\pi }_{2}{\mathcal{N}}\left(0,{\sigma }_{2}^{2}\right)+\ldots +{\pi }_{L}{\mathcal{N}}\left(0,{\sigma }_{L}^{2}\right)$$

where $\left\{{\pi }_{0},{\pi }_{1},{\pi }_{2},\ldots ,{\pi }_{L}\right\}$ are the mixture proportions and $\left\{{\sigma }_{1}^{2},{\sigma }_{2}^{2},\ldots ,{\sigma }_{L}^{2}\right\}$ are the mixture-specific variances and *δ*_0_ is a discrete probability mass at zero.

We further constrain the prior by assuming a single parameter representing the total variance explained by the effects *σ*^2^, and the component-specific variances are proportional to *σ*^2^, that is

4 $$\left[\begin{array}{c}{\sigma }_{1}^{2}\\ {\sigma }_{2}^{2}\\ \vdots \\ {\sigma }_{L}^{2}\end{array}\right]={\sigma }^{2}\left[\begin{array}{c}{C}_{1}\\ {C}_{2}\\ \vdots \\ {C}_{L}\end{array}\right]$$

with $\left\{{C}_{1},{C}_{2},\ldots ,{C}_{L}\right\}$ being constants.

Our main contribution consists of allowing different subsets of Θ to have specific *σ*^2^ and *π* parameters, our contention is that in cases where one data source’s effect are in a different scale than on the other, the extra degrees of freedom will allow to better resolve the smaller effects, whereas the group specific variance parameters will pool information of the effects within a group and keep the model identifiable. In our case, we assign to the genetic effects *β*_*G*_ a set of mixture variances ${C}_{G}=\left\{0.0001,0.001,0.01\right\}$, a proportion parameter *π*_*G*_ and variance parameter ${\sigma }_{G}^{2}$. We assign to the methylation probes, age, and sex effects $\phi =\left\{{\beta }_{cpg},\alpha ,\gamma \right\}$ the prior variances ${C}_{\phi }=\left\{0.01,0.1,1\right\}$ and parameters ${\pi }_{\phi },{\sigma }_{\phi }^{2}$

The rest of the model follows the prior hierarchy of ref. ^31^ but with additional parameters for groups *G* and *ϕ*.

5 $${\pi }_{G} 	\sim {\rm{Dirichlet}}\ \left({{\bf{p}}}_{G}\right)\\ {\pi }_{\phi } 	\sim {\rm{Dirichlet}}\ \left({{\bf{p}}}_{\phi }\right)\\ {\sigma }_{G}^{2} 	\sim {\rm{Inv}}-{\rm{Scaled}}{\chi }^{2}\left({v}_{0},{s}_{0}^{2}\right)\\ {\sigma }_{\phi }^{2} 	\sim {\rm{Inv}}-{\rm{Scaled}}{\chi }^{2}\left({v}_{0},{s}_{0}^{2}\right)\\ {\sigma }_{\epsilon }^{2} 	\sim {\rm{Inv}}-{\rm{Scaled}}{\chi }^{2}\left({v}_{0},{s}_{0}^{2}\right)$$

with the respective hyperparameters $\left\{{{\bf{p}}}_{G},{{\bf{p}}}_{\phi },{{\rm{v}}}_{0},{s}_{0}^{2}\right\}$ such that the prior distributions are weakly informative, ${{\bf{p}}}_{G}={{\bf{p}}}_{\phi }=\left(1,1,1\right),{{\rm{v}}}_{0}={{\rm{s}}}_{0}^{2}=0.001$.

### Model inference

Inference of the probabilistic model follows a Gibbs sampling algorithm. Here, the joint posterior probability density of parameters $\left(\Theta ,\mu ,{\sigma }_{\epsilon }^{2},{\sigma }_{G}^{2},{\sigma }_{\phi }^{2},{\pi }_{G},{\pi }_{\phi }\right)$ conditioned on observed phenotype **y** and observed covariates ${\bf{Z}}=\left[\,{{\bf{X}}}_{G}\,{{\bf{X}}}_{cpg}\,age\,sex\,\right]$ is denoted as $p\left(\Theta ,\mu ,{\sigma }_{\epsilon }^{2},{\sigma }_{G}^{2},{\sigma }_{\phi }^{2},{\pi }_{G},{\pi }_{\phi }| {\bf{Z}},{\bf{y}}\right)$ and decomposed according to the conditional distributions over each parameter:

6 $$p\left(\Theta ,\mu ,{\sigma }_{\epsilon }^{2},{\sigma }_{G}^{2},{\sigma }_{\phi }^{2},{\pi }_{G},{\pi }_{\phi }| Z,{\bf{y}}\right) 	\approx p\left(\mu | \Theta ,{\sigma }_{\epsilon }^{2}Z,{\bf{y}}\right)\\ 	\quad \times p\left(\Theta | {\sigma }_{G}^{2},{\sigma }_{\phi }^{2},\mu ,{\sigma }_{\epsilon }^{2},Z,{\bf{y}}\right)\\ 	\quad \times p\left({\sigma }_{G}^{2}| {\beta }_{G},{\pi }_{G}\right)\\ 	\quad \times p\left({\pi }_{G}| {\beta }_{G},{\sigma }_{G}^{2}\right)\\ 	\quad \times p\left({\sigma }_{\phi }^{2}| {\beta }_{\phi },{\pi }_{\phi }\right)\\ 	\quad \times p\left({\pi }_{\phi }| {\beta }_{\phi },{\sigma }_{\phi }^{2}\right)\\ 	\quad \times p\left({\sigma }_{\epsilon }^{2}| \mu ,\Theta ,Z,{\bf{y}}\right).$$

Given the prior distributions presented in Eq. 2, the conditional distributions are:

7 $$ p\left(\mu | \Theta ,{\sigma }_{\epsilon }^{2}Z,{\bf{y}}\right)\propto {\mathcal{N}}\left(\frac{\mathop{\sum }\nolimits_{i = 1}^{N}\left({{\bf{y}}}_{i}-{{\bf{Z}}}_{{\bf{i}}}\Theta \right)}{N},\frac{{\sigma }_{\epsilon }^{2}}{N}\right)$$

8 $$p\left({\sigma }_{G}^{2}| {\beta }_{G},{\pi }_{G}\right)\propto {\rm{Inv}}-{\rm{Scaled}}{\chi }^{2}\left({m}_{G}+{{\rm{v}}}_{0},\frac{{m}_{G}\mathop{\sum }\nolimits_{i = 1}^{{M}_{G}}{\beta }_{Gi}+{{\rm{v}}}_{0}{S}_{0}^{2}}{{{\rm{v}}}_{0}+{m}_{G}}\right)$$

9 $$p\left({\pi }_{G}| {\beta }_{G},{\sigma }_{G}^{2}\right)\propto {\rm{Dirichlet}}\left({{\bf{p}}}_{\phi }+\#{{\bf{K}}}_{G}\right)$$

10 $$p\left({\sigma }_{\phi }^{2}| {\beta }_{\phi },{\pi }_{\phi }\right)\propto {\rm{Inv}}-{\rm{Scaled}}{\chi }^{2}\left({m}_{\phi }+{{\rm{v}}}_{0},\frac{{m}_{\phi }\mathop{\sum }\nolimits_{i = 1}^{{M}_{G}}{\beta }_{\phi i}+{{\rm{v}}}_{0}{S}_{0}^{2}}{{{\rm{v}}}_{0}+{m}_{\phi }}\right)$$

11 $$p\left({\pi }_{\phi }| {\beta }_{\phi },{\sigma }_{\phi }^{2}\right)\propto {\rm{Dirichlet}}\left({{\bf{p}}}_{\phi }+\#{{\bf{K}}}_{\phi }\right)$$

12 $$p\left({\sigma }_{\epsilon }^{2}| \mu ,\Theta ,Z,{\bf{y}}\right)\propto {\rm{Inv}}-{\rm{Scaled}}{\chi }^{2}\left({{\rm{v}}}_{0}+N,\frac{\mathop{\sum }\nolimits_{i = 1}^{N}{\left({{\bf{y}}}_{i}-\mu -{{\bf{Z}}}_{i}\Theta \right)}^{2}+{{\rm{v}}}_{0}{S}_{0}^{2}}{{{\rm{v}}}_{0}+N}\right)$$

where *m*_*G*_ and *m*_*ϕ*_ are the number of markers in each respective category in a sample and *#***K**_*G*_, *#***K**_*ϕ*_ are vectors that contain the number of markers in each mixture for the respective categories.

### Residual updating algorithm

The most computationally expensive step of sampling from the distribution in Eq. 2 involves drawing from the conditional distribution

13 $$p\left(\Theta | {\sigma }_{G}^{2},{\sigma }_{\phi }^{2},\mu ,{\sigma }_{\epsilon }^{2},Z,{\bf{y}}\right)$$

if conditioned on the Markov blanket of effects Θ, the distribution is a multivariate normal with mean **m** and co-variance Σ such that

14 $${\bf{m}}=\Sigma {{\bf{Z}}}^{T}{\bf{y}}$$

15 $$\Sigma ={\sigma }_{\epsilon }^{-2}{\left({{\bf{Z}}}^{T}{\bf{Z}}+\frac{{\sigma }_{\epsilon }^{2}}{{\sigma }_{l}^{2}}{\bf{I}}\right)}^{-1}$$

with ${\sigma }_{l}^{2}$ being the mixture-specific variance and the residual’s variance ${\sigma }_{\epsilon }^{2}$. Inverting matrix Σ is of complexity ${\mathcal{O}}\left({({M}_{G}+{M}_{\phi })}^{3}\right)$. If we use the properties of multivariate Gaussian distributions, we can decompose $p\left(\Theta | {\sigma }_{G}^{2},{\sigma }_{\phi }^{2},\mu ,{\sigma }_{\epsilon }^{2},Z,{\bf{y}}\right)\propto \mathop{\prod }\nolimits_{i = 1}^{{M}_{G}+{M}_{\phi +2}}p\left({\Theta }_{i}| {\Theta }_{\backslash i}{\sigma }_{G}^{2},{\sigma }_{\phi }^{2},\mu ,{\sigma }_{\epsilon }^{2},Z,{\bf{y}}\right)$, where Θ_\*i*_ represents all the effects except effect Θ_*i*_. Then each individual update consists of:

16 $$p\left({\Theta }_{i}| {\Theta }_{\backslash i}{\sigma }_{G}^{2},{\sigma }_{\phi }^{2},\mu ,{\sigma }_{\epsilon }^{2},Z,{\bf{y}}\right)\propto {\mathcal{N}}\left({\mu }_{i},{\Sigma }_{li}\right)$$

with

17 $${\mu }_{i} 	=\mathop{\Sigma }\limits_{i}{{\bf{Z}}}_{i}^{T}\left({\bf{y}}-\mu -{{\bf{Z}}}_{\backslash i}{\Theta }_{\backslash i}\right)\\ \mathop{\Sigma }\limits_{li} 	={\sigma }_{\epsilon }^{-2}{\left({{\bf{Z}}}_{i}^{T}{{\bf{Z}}}_{i}+\frac{{\sigma }_{\epsilon }^{2}}{{\sigma }_{l}^{2}}\right)}^{-1}.$$

This obviates the necessity of inverting matrix Σ, if in addition we keep in memory the vector of residuals *ϵ* = **y** − *μ* − **Z**Θ, then we can compute efficiently **y** − *μ* − **Z**_*i*_Θ_*i*_ by the update ${\bf{y}}-\mu -{{\bf{Z}}}_{i}{\Theta }_{i}=\epsilon +{{\bf{Z}}}_{i}{\Theta }_{i}=\tilde{{\bf{y}}}$, thus sampling from the joint distribution with a complexity ${\mathcal{O}}\left({M}_{G}+{M}_{\phi }\right)$. Mixing and convergence issues that may arise in this formulation have been shown to be alleviated by randomly choosing an effect Θ_*i*_ to update, as seen in successful implementations of the algorithm^31^.

**Table:** 

**Algorithm 1** Algorithm for sampling over the posterior distribution $p\left(\mu ,\beta ,\epsilon ,{\sigma }_{\epsilon },\theta \right)$, each sample (*μ*, *β*, *ϵ*, *σ*_*ϵ*_, *θ*) is stored in a synchronized queue for a consumer thread to store in disk. ${{\bf{X}}}_{marke{r}_{j}}$ represents column of **X** corresponding to the column *j* of the vector marker. Given that marker is shuffled before sampling the effects, this is equivalent to permuting the order of the effects to be sampled.
Input: genotype matrix **X**_*G*_, methylation probe matrix **X**_*cpg*_, age and sex, vector of trait measurements **y**, prior hyperparameters $\{{{\bf{p}}}_{G},{{\bf{p}}}_{\phi },{{\rm{v}}}_{0},{s}_{0}^{2}\}$, number of iterations I.
Output: mean *μ*, effects vector $\Theta =\left\{{\beta }_{M},{\beta }_{G},\alpha ,\gamma \right\}$, residual vector *ϵ*, residuals variance ${\sigma }_{\epsilon }^{2}$, and posterior parameters, ${\sigma }_{G}^{2},{\sigma }_{\phi }^{2}$
1. Initialize $$\Theta ,\mu ,{\sigma }_{\epsilon }^{2},{\sigma }_{G}^{2},{\sigma }_{\phi }^{2},{\pi }_{G},{\pi }_{\phi }$$
2. *effects* = $$(1\ldots {M}_{G},({M}_{G}+1)\ldots ({M}_{G}+{M}_{\phi }),({M}_{G}+{M}_{\phi }+1),({M}_{G}+{M}_{\phi }+2))$$
3. set $${\bf{Z}}=[\,{{\bf{X}}}_{G}\,\,{{\bf{X}}}_{cpg}\,\,age\,\,sex\,]$$
4. *ϵ* = **y** − *μ* − **Z**Θ
5. For i in 1...I
(a) sample *μ* =
(b) *shuffle* $$\left(effects\right)$$
(c) For j in $$1\ldots ({M}_{G}+{M}_{\phi }+2)$$
i. $$\,\,\,\tilde{y}=\epsilon +{{\bf{Z}}}_{effec{t}_{j}}{\Theta }_{effec{t}_{j}}$$
ii. Sample $${\Theta }_{effec{t}_{j}}$$
iii. $$\,\epsilon =\tilde{y}-{{\bf{Z}}}_{effec{t}_{j}}{\Theta }_{effec{t}_{j}}$$
(d) sample $${\sigma }_{\epsilon }^{2}$$
(e) sample $${\sigma }_{G}^{2}$$
(f) sample $${\sigma }_{\phi }^{2}$$
(g) *enqueue* (*μ*, *β*, *ϵ*, *σ*_*ϵ*_, *θ*)

### Drawing from the mixtures

To select the mixture *l* from which to draw the effect Θ_*i*_, we must evaluate the likelihood ratio between all the mixtures. Using the log-likelihood ${\mathcal{L}}$, this amounts to:

18 $${\mathcal{L}}\left(i,l\right)\left\{\begin{array}{l}\mathrm{log}\,{\pi }_{0}\\ \mathrm{log}\,{\pi }_{\mathrm{k}}-\frac{1}{2}\left|\mathrm{N}\mathrm{log}\,{\Sigma }_{\mathrm{li}}\right|-\frac{1}{2}{\mu }_{\mathrm{i}}^{2}{\Sigma }_{\mathrm{li}}^{-1}\quad \mathrm{rest}\end{array}\right.$$

Finally, the probability of drawing effect Θ from mixture *l* is given by:

19 $$p\left(l\right)=\frac{1}{\mathop{\sum }\nolimits_{j = 1}^{L}\exp [{\mathcal{L}}\left(i,j\right)-{\mathcal{L}}\left(i,l\right)]}$$

### Software implementation

The algorithm was implemented in C++−11.0, with the help of the templated matrix algebra library Eigen^32,33^ and Intel’s Threading Building Blocks library^34^. Source code available in https://github.com/ctggroup/BayesRRcmd.

### Simulation study of methylation data

We use a generative model of methylation levels as in ref. ^12^, where the matrix ${\bf{O}}\in {{\mathbb{R}}}^{{\rm{N}}\times {\rm{M}}}$ represents the methylation levels for M probes in N individuals. This matrix can be decomposed in a matrix of K cell proportions for N individuals, which we denote ${\bf{R}}\in {{\mathbb{R}}}^{{\rm{N}}\times {\rm{K}}}$, and a matrix of cell-specific methylation levels ${\bf{S}}\in {{\mathbb{R}}}^{{\rm{K}}\times {\rm{M}}}$. The decomposition assumes i.i.d. observation noise such that

20 $${\bf{O}}={\bf{M}}+{{\vartheta }}$$

where the observation noise ${\vartheta }_{i,j} \sim {\mathcal{N}}\left(0,{\nu }_{j}^{2}\right)$, such that $i\in \left(1\ldots N\right)$ and $j\in \left(1\ldots M\right).$ The methylation level matrix *M* is decomposed in

21 $${\bf{M}}={\bf{RS}}$$

For the simulation, each row *i* of **R** is distributed according to

22 $${r}_{i} \sim {\rm{Dirichlet}}\ \left(\theta \right)$$

and each of the methylation levels ${s}_{ij}\in {\mathbb{R}}$ are distributed as follows for the differentially methylated probes (DMP)

23 $${s}_{ij} \sim {{\mathcal{N}}}_{\left(0,1\right)}\left(0,{\tau }^{2}\right)$$

being ${{\mathcal{N}}}_{\left(0,1\right)}$ the truncated normal distribution with support $\left[0,1\right]$. For the non-DMP we set the methylation levels to a base value

For these simulations we relied on software kindly provided by the authors^12^. We performed a slight modification to be able to change the variance of the cell proportions matrix **P**. For each simulation we generated a matrix of *N* = 2000 and *M* = 103,638. The simulation parameters were in accordance to^12^:

- Proportion of differentially methylated probes (*p*).
- Variance of the DMPs(*τ*).
- Variance of the cell proportions (*sp*).
- Variance of the measurement noise (*ϑ*)

We assume 100 probes exert an effect over phenotypic trait **y**, thus for each probe selected we denote *β*_*d*_ as the effect corresponding to a probe, and their respective effects are drawn from

24 $${\beta }_{d} \sim {\mathcal{N}}\left(0,{\sigma }_{\beta }^{2}\right)$$

the rest of the effects are assign a value of 0. Finally, we simulate **y** from the linear model in which phenotype is determined by effects over the noiseless methylation matrix: **y** = **MB** + *ϵ* with ${\sigma }_{\epsilon }^{2}=$0.6 and ${\sigma }_{\beta }^{2}=\frac{0.5}{100}$ where the variance explained (VE) by the probes amounts to 0.6. For each of the following scenarios, 15 simulations were performed:

- $$p\in \left\{0.1,0.3,0.7,0.9\right\},\tau =0.07$$, *sp* = 1000,*ϑ* = 0.01
- $$p=0.15,\tau \in \left\{0.01,0.03,0.05,0.09\right\},sp=1000$$, *ϑ* = 0.01
- $$p=0.15,\tau =0.07,sp\in \left\{0.001,0.1,1,10,1000\right\}$$, *ϑ* = 0.01
- $p=0.15,\tau =0.07,sp=1000,\vartheta \in \left\{0.01,0.025,0.05,0.075\right\}$, for this case the simulated model consists of effects over the noisy methylation matrix **Oy** = **OB** + *ϵ*.

which amounts to 255 simulated data sets in total.

For each of the 270 simulations, we ran our method for the observed phenotype **y** centered and scaled to variance 1, the observed methylation matrix **O** as inputs and with mixture variances (0.1, 0.01, 0.001, 0.0001), for 20,000 samples with 10,000 samples of burn-in after which a thinning of 10 samples was used to select samples of the posterior for the simulation. We selected trait-associated probes as those that were in the model in  >95% of the posterior samples, which we define as 95% posterior IP.

We used the following metrics to assess model performance: the correlation between true effects and estimates ($\rho (\beta ,\hat{\beta })$), the slope of a regression of the estimates on the true effects (${\beta }_{\hat{\beta } \sim \beta }$), the number of genome-wide significant probes identified (loci), the MSE, the MSE of the genome-wide significant probes (*MSE*_*sig*_), the false discovery rate (FDR), the norm of the correlation vector between a individual-level predictor made from the probe effects and the cell-type proportions ($| | \rho ({\bf{R}},\hat{{\bf{g}}})| |$), the correlation between the first principal component of the probe data and the difference between the estimated and true effect (∣∣*ρ*(**P**, **R**)∣∣) and the phenotypic variance attributable to the probes (${\sigma }_{cg}^{2}$). These statistics were obtained for the simulations and shown in Supplementary Figs. 1–3.

### Competing methods

For comparisons with our method we chose the common EWAS methodology, which is derived from the GWAS methodology. For each simulation replicate we ran the following models:

*Single-probe least squares regression (GWAS)*: We conducted a set of 103,638 linear regressions using the function lm() from R version 3.4.2, and accepted the effect sizes whose *p* value (*t* test) was <0.05/103638 (Bonferroni correction).

*Single-probe least squares regression with sparse latent factors (ReFACTor)*: Using the approach outlined in ref. ^12^, we selected five sparse components (one for each cell type in the simulation) and estimated probe associations conditional upon these one-by-one. We selected associated probes whose *p* value (*t* test) was less than 0.05/103638.

*Single-probe mixed linear model association analysis (OSCA)*: Using the approach outlined in^13^, we used a linear mixed effects model where probe values are used to calculate a co-variance matrix, which is then used in a REML approach to estimate the proportion of phenotypic variance attributable to the probes, and conditional upon this probe effects are estimated one-by-one. Although this estimates probe associations conditional on the co-variance matrix, it does not account for genome-wide co-variance across probes. We selected associated probes whose *p* value (*t* test) was <0.05/103,638.

*Multi-probe penalized regression with latent factors (LFMM)*: Using the approach outlined in ref. ^11^, we ran LASSO and ridge regression with 10-fold cross-validation using the default settings (with five latent factors, one for each cell type in the simulation), whereas also fitting latent factors within the model that are intended to control for cell-type proportion confounding. We used the lfmm_function and selected probes based on the calibrated *p* value (*t* test) whose *p* value (*t* test) was <0.05/103,638.

*Multi-probe penalized regression without latent factors (glmnet)*: We ran LASSO and ridge regression with 10-fold cross-validation using the default settings of package glmnet^16^ version 2.0-16. Previous experiments suggested that best performance was achieved by leaving the phenotype vector **y** un-scaled.

### Simulations of genotype and methylation effects

We simulated a methylation matrix **M** with parameters *p* = 0.15, *τ* = 0.07, *sp* = 1000, *ϑ* = 0.01 as above, with the same number of SNPS as the methylation matrix (103,638). Then, we generated a genotype matrix ${\bf{X}}\in {{\mathbb{R}}}^{1000\times 103638}$. For each of the DMPS with non-zero effects in **M** we select column *j* of *X* to have a correlated genotype by sampling its elements from the distribution:

25 $${X}_{ij} \sim Binomial \left(2,\frac{1}{1+\exp (10-c* {M}_{ij})}\right)$$

by sampling c from an uniform distribution between 20 and 25 for each column, we achieve a pairwise genotype-methylation correlation between 0 and 0.6 for a genotype column and a corresponding column of methylation profiles (for those methylation probes with non-zero effects).

Having both matrices **O** (the noisy observations over matrix **M**) and **X** centered and scaled, we generated 100 methylation effects ${\beta }_{cg} \sim {\mathcal{N}}\left(0,\frac{0.5}{100}\right)$, 100 large genotypic effects ${\beta }_{g} \sim {\mathcal{N}}\left(0,\frac{0.2}{100}\right)$ and 900 small genotypic effects ${\beta }_{g} \sim {\mathcal{N}}\left(0,\frac{0.1}{900}\right)$. Thus, the model for the simulated genotype is **y**   **O***β*_*cg*_ + **X***β*_*g*_ + *ϵ*, with $\epsilon \sim {\mathcal{N}}\left(0,0.2\right)$.

We repeated the process 15 times to have 15 simulated data sets. For each of the simulated data sets we ran our method for 200,00 samples, with a burn-in of 100,00 and a thinning of 10 samples. The mixture variances were set to (0.1, 0.01, 0.001, 0.0001) for methylation effects and to (0.01, 0.001, 0.0001, 0.00001) for genotype effects. We compared out approach with LASSO and Ridge regression implemented in glmnet^16^, with a baseline of single marker regression (GWAS) where we first adjusted the phenotype by the first 10 principal components of the genotype matrix and then regressed the residuals against the scaled methylation matrix. The methods were compared over the estimation of the true genetic and epigenetic VE and ability to estimate the true effects. Results shown in Supplementary Fig. 4.

### Generation Scotland

Generation Scotland: the Scottish Family Health Study is a large population-based, family-structured cohort of over 24,000 individuals aged 18–99 years. The study baseline took place between 2006 and 2011 and included detailed cognitive, physical, and health questionnaires, along with sample donation for genetic and biomarker data. DNA methylation data from whole blood was obtained on a subset of ~10,000 participants. The Illumina HumanMethylationEPIC Bead Chips array was used to measure methylation and quality control details have been reported previously^29^. In briefly, outliers based on the visual inspection of methylated to unmethylated log intensities were excluded, along with poorly performing probes and samples, and sex mismatches (predicted based on genetics versus questionnaire data) yielding an analysis data set of 9448. As reported in McCartney et al.^29^, further filtering was performed to exclude non-autosomal CpG sites and sites that were exclusive to the EPIC array. This allowed for the predictors to be applied to data sets that collected DNA methylation using an earlier version of the Illumina arrays (450 k array) giving a total of 370,262 probes.

After the quality control steps described above, we integrated the SNP marker and methylation matrices, along with the log-transformed age and the sex of the individuals (encoded as 1 for Female). All matrices and phenotypes were centered and scaled to variance 1. The data were used as input for our Bayesian model, with parameters (0.0001, 0.001, 0.01) for genetic effects mixtures variances and (0.01, 0.1, 1) for the epigenetic effects, age, and sex. Four chains for each trait with different starting values were executed. We assessed the convergence of the hyperparameters ${\sigma }_{\epsilon }^{2},{\sigma }_{G}^{2},{\sigma }_{\phi }^{2}$ through the Geweke test^35^ and the $\hat{R}$ criteria^36^, with the help of the R package ggmcmc^37^, see Supplementary Figs. 5–14. As result, the algorithm yielded a set of samples over the posterior distribution of effects conditioned on the observed phenotype, the genetic and epigenetic probes and controlled by age and sex. We further scaled in each sample the hyperparameters ${\sigma }_{\epsilon }^{2},{\sigma }_{G}^{2}$, and ${\sigma }_{\phi }^{2}$ by dividing each one by their sum (${\sigma }_{\epsilon }^{2}+{\sigma }_{G}^{2}+{\sigma }_{\phi }^{2}$). The posterior distribution is summarized in Supplementary Table 1 for BMI and Supplementary Table 2 for smoking. IP were computed by counting the times a probe is present in the model (in any of the mixtures) and divided by the total number of posterior samples. We performed the same procedure but adding the first 20 PCs of the DNA methylation matrix, the first 20 PCs of the genotype matrix along with the cell composition of the samples as extra 46 covariates. We regressed the mean of the posterior effect sizes of the model without these covariates against the model with these covariates. The mean effect sizes are practically identical for BMI and smoking, with an *R*^2^ of 0.97 slope of 0.99 for BMI and *R*^2^ of 0.99 slope of 1 for smoking.

We then contrasted the variance explained by the first five PCs of the probes with 95% PIP in BMI and smoking, to predict their respective phenotypes. We found that these first five PCs in BMI probes do not have predictive power for BMI, thus, this suggests that the main axis of variation of these probes with 95% PIP do not explain as much of the phenotype as the whole set of probes with 95% PIP for BMI. For smoking the five PCs derived from the probes with 95% PIP for the same phenotype explain ~44% of the variance. We further verified that the predictive power of the 46 covariates (genetic and methylation PCs, along with cell-count effects) explain 7% variance in BMI and 10% variance in smoking. We observed that the first PC for the probes with 95% PIP for BMI is highly correlated with cell counts (Supplementary Fig. 19), the fact that these PCs do not explain much variance in BMI suggests that these correlations are not meaningful for predicting the phenotype. For smoking, it is clear that the PCs of the probes with 95% PIP and the cell counts are weakly correlated (Supplementary Fig.  20), thus the predictive power for these PCs seems to be not aligned to cell-counts effects. These analyses suggest that for BMI the probes with 95% PIP do not explain together as much variance as the analogous probes for smoking, that the variance explained by the probes with 95% PIP is almost the same as the variance explained by their PCs and that in both cases, their predictive power seems to be independent to their correlation with cell counts.

These results, stability of estimates even after adjusting for confounders, and predictive power independent to cell counts, support our conclusions that the learned model’s effect carry information over the traits of interest.

### Enrichment analysis

Probes were associated to their respective gene ENTREZ identifiers using the R packages IlluminaHumanMethylation450kanno.ilmn12.hg19^38^ and biomaRT^39^. We provide a list of each gene with IP >5% for BMI Supplementary Table 4, and for smoking Supplementary Table 5.

Then, we associated the mapped genes with their respective terms in the Gene Onthology (GO) using the R package^40^. With these probe-terms associations we computed enrichment as defined by:

26 $$enrichment=\frac{p.PVE}{p.Term}$$

with *p*. *PVE* being the proportion of variance explained by probes associated with the term, having *β*_*Term*_ being the effects associated with the term and being *β*_*Model*_ the effects in the model (that is, those which are not coming from the spike at zero) in the current sample, we have

27 $$p.PVE=\frac{{\sum }^{Term}{\beta }_{Term}^{2}}{{\sum }^{Model}{\beta }_{Model}^{2}}$$

and *p*.*Term* being the proportion of probes mapping to the term among all the probes mapping to a term in the current sample. Having *#probes*_*Term*_ being the number of probes mapping to a term and *#probes*_*Model*_ the number of probes in the model in the current sample, we have

28 $$p.Term=\frac{\#probe{s}_{Term}}{\#probe{s}_{Model}}$$

We also computed the IP for a term by counting the times a term appears in the model and dividing by the number of samples. Finally, given that we have a posterior distribution over enrichment values, we adopt the ROPE decision rule^23^, for which, we accept the hypothesis that a term is significantly over/under-enriched if 95% of the posterior mass for the enrichment value is outside the interval (0.5, 1.5) and the term has an IP  >95%. Significantly enriched GO terms are presented in the Supplementary Information.

### Estimates for replication

For both BMI and smoking, the posterior samples over effects where averaged and associated to their respective probe and SNP identities. For each replication cohort, a predictor was built by multiplying the posterior mean effects by the corresponding centered and scaled genetic and epigenetic marker readings, and predictive ability measured over the scaled and centered cohort trait was measured using the *R*^2^ statistic.

### Lothian Birth Cohort 1936

The Lothian Birth Cohort 1936 is a longitudinal study of aging^41^. It follows 1091 members of the 1947 Scottish Mental Survey, who were recontacted in later life, when they were living in the Edinburgh area of Scotland. The cohort members were all born in 1936 and have been assessed for a wide variety of health and lifestyle outcomes at ages 70, 73, 76, 79, and 82 years. DNA has been collected at each clinical visit. In the present study, we considered DNA methylation data (Illumina 450 k array) from whole blood, taken at mean age 70, for analysis. Details of the collection and processing of the data have been reported previously^29^. In brief, after quality control to remove poorly performing methylation sites, samples, and individuals with mismatching genotypes or predicted sex, a sample of 906 individuals was available for prediction analysis. The genotype and methylation matrices were processed as with GS, given that the posterior effect sizes for age and sex were equal to zero for both traits, they were not included.

### The UK adult twin Registry

The TwinsUK registry consists of over 14,000 research volunteer twin participants from the United Kingdom who have joined since 1992, with equal numbers of same-sex monozygotic and dizygotic twin pairs who are predominately female (84%). Data are collected through longitudinal questionnaires and clinical visits. The registry collects biological samples and further data collected through analysis of biological samples. DNA methylation profiles were generated using the Infinium HumanMethylation450 BeadChip in adipose tissue biopsies and whole-blood samples from TwinsUK participants. Adipose tissue DNA methylation data were generated from subjects who were free from severe diseases, as previously described^42^. Whole-blood DNA methylation profiles have previously been described^43^. Additional data processing steps for this project included use of ENmix^44^ for quality control and minfi^45^ to exclude samples with median methylated and unmethylated signals below 10.5. After data-quality control, covariate assessments, and sample processing, downstream analyses were carried out in 540 adipose (mean age 59, age range 28–85, 100% female) and 977 whole blood (mean age 58, age range 19–82, 97% female) TwinsUK samples. Ethical approval was granted by the National Research Ethics Service London-Westminster, the St Thomas’ Hospital Research Ethics Committee (EC04/015 and 07/H0802/84). All research participants provided written informed consent prior to taking part in the study.

### Avon Longitudinal Study of Parents and Children

Samples were drawn from the Avon Longitudinal Study of Parents and Children^46^,^47^. Blood from 1018 mother–child pairs (children at three time points and their mothers at two time points) were selected for analysis as part of the Accessible Resource for Integrative Epigenomic Studies (ARIES, http://www.ariesepigenomics.org.uk/)^48^. Following DNA extraction, samples were bisulphite converted using the Zymo EZ DNA Methylation kit (Zymo, Irvine, CA, USA). Following conversion, genome-wide methylation was measured using the Illumina Infinium HumanMethylation450 (HM450) BeadChip. The arrays were scanned using an Illumina iScan, with initial quality review using GenomeStudio. ARIES was preprocessed and normalized using the meffil R package^49^. ARIES consists of 5469 DNA methylation profiles obtained from 1022 mother–child pairs measured at five time points (three time points for children: birth, childhood, and adolescence; and two for mothers: during pregnancy and at middle age). Low-quality profiles were removed from further processing, and the remaining 4593 profiles were normalized using the Functional Normalization algorithm^50^ with the top 10 control probe principal components. Full details of the preprocessing and normalization of ARIES have been described previously^49^.

### Reporting summary

Further information on research design is available in the Nature Research Reporting Summary linked to this article.

## Supplementary information

Supplementary Information

Peer Review File

Reporting Summary

## Data Availability

Data are available upon request from the cohort authors with appropriate research agreements.

## Code availability

BayesRR R implementation and full open source code is available at: https://github.com/ctggroup/bayesRRcpp. BayesRR is also implemented as part of the command line tool BayesR+ at: https://github.com/ctggroup/bayesRRcmd. Simulation scripts and post-processing scripts can be found here: https://github.com/ctggroup/BEpigenetics.
