# Author Correction: Bayesian reassessment of the epigenetic architecture of complex traits

**DOI:** 10.1038/s41467-020-19099-9

**Published:** 2020-10-09

**Authors:** Daniel Trejo Banos, Daniel L. McCartney, Marion Patxot, Lucas Anchieri, Thomas Battram, Colette Christiansen, Ricardo Costeira, Rosie M. Walker, Stewart W. Morris, Archie Campbell, Qian Zhang, David J. Porteous, Allan F. McRae, Naomi R. Wray, Peter M. Visscher, Chris S. Haley, Kathryn L. Evans, Ian J. Deary, Andrew M. McIntosh, Gibran Hemani, Jordana T. Bell, Riccardo E. Marioni, Matthew R. Robinson

**Affiliations:** 1 Department of Computational Biology, University of Lausanne, Lausanne, Switzerland; 2 Centre for Genomic and Experimental Medicine, Institute of Genetics and Molecular Medicine, University of Edinburgh, Edinburgh, UK; 3 MRC Integrative Epidemiology Unit, University of Bristol, Bristol, UK; 4 Population Health Sciences, Bristol Medical School, University of Bristol, Bristol, UK; 5 Department of Twin Research and Genetic Epidemiology, King’s College London, London, UK; 6 Institute for Molecular Bioscience, University of Queensland, Brisbane, QLD Australia; 7 MRC Human Genetics Unit, Institute of Genetics and Molecular Medicine, University of Edinburgh, Edinburgh, UK; 8 Centre for Cognitive Ageing and Cognitive Epidemiology, University of Edinburgh, Edinburgh, UK; 9 Department of Psychology, University of Edinburgh, Edinburgh, UK; 10 Division of Psychiatry, University of Edinburgh, Royal Edinburgh Hospital, Edinburgh, UK; 11 Institute of Science and Technology Austria, Klosterneuburg, Austria

**Keywords:** Statistical methods, Epigenomics, Predictive markers

Correction to: *Nature Communications* 10.1038/s41467-020-16520-1, published online 8 June 2020.

The original version of this Article contains an error in Fig. 3 in which panel B was inadvertently duplicated from panel A. This has been corrected in both the PDF and HTML versions of the Article.

