# Supplementary material for: Bayesian reassessment of the epigenetic architecture of complex traits
Source: Nat Commun. 2020 Jun 8;11:2865. doi: 10.1038/s41467-020-16520-1 (PMC7280277; doi:10.1038/s41467-020-16520-1)
Supplement: Supplementary file 3 — Reporting Summary [file 41467_2020_16520_MOESM3_ESM.pdf]

nature research

Corresponding author(s): Trejo-Banos, Robinson

Last updated by author(s): Apr 24, 2020

Reporting Summary

Nature Research wishes to improve the reproducibility of the work that we publish. This form provides structure for consistency and transparency in reporting. For further information on Nature Research policies, see Authors & Referees and the Editorial Policy Checklist.

Statistics

For all statistical analyses, confirm that the following items are present in the figure legend, table legend, main text, or Methods section.

n/a	Confirmed	
☐	☒	The exact sample size (*n*) for each experimental group/condition, given as a discrete number and unit of measurement
☐	☒	A statement on whether measurements were taken from distinct samples or whether the same sample was measured repeatedly
☐	☒	The statistical test(s) used AND whether they are one- or two-sided<br>*Only common tests should be described solely by name; describe more complex techniques in the Methods section.*
☐	☒	A description of all covariates tested
☐	☒	A description of any assumptions or corrections, such as tests of normality and adjustment for multiple comparisons
☐	☒	A full description of the statistical parameters including central tendency (e.g. means) or other basic estimates (e.g. regression coefficient) AND variation (e.g. standard deviation) or associated estimates of uncertainty (e.g. confidence intervals)
☐	☒	For null hypothesis testing, the test statistic (e.g. *F*, *t*, *r*) with confidence intervals, effect sizes, degrees of freedom and *P* value noted<br>*Give P values as exact values whenever suitable.*
☐	☒	For Bayesian analysis, information on the choice of priors and Markov chain Monte Carlo settings
☐	☒	For hierarchical and complex designs, identification of the appropriate level for tests and full reporting of outcomes
☐	☒	Estimates of effect sizes (e.g. Cohen's *d*, Pearson's *r*), indicating how they were calculated

Our web collection on statistics for biologists contains articles on many of the points above.

Software and code

Policy information about availability of computer code

Data collection	BayesRR R implementation and full open source code is available at: \url{https://github.com/ctggroup/bayesRRcpp}.<br><br>BayesRR is also implemented as part of the command line tool BayesR+ at:<br>\url{https://github.com/ctggroup/bayesRRcmd}.<br><br>Simulation scripts and post-processing scripts can be found here: \url{https://github.com/ctggroup/BEpigenetics}.
Data analysis	BayesRR R implementation and full open source code is available at: \url{https://github.com/ctggroup/bayesRRcpp}.<br><br>BayesRR is also implemented as part of the command line tool BayesR+ at:<br>\url{https://github.com/ctggroup/bayesRRcmd}.<br><br>Simulation scripts and post-processing scripts can be found here: \url{https://github.com/ctggroup/BEpigenetics}.

For manuscripts utilizing custom algorithms or software that are central to the research but not yet described in published literature, software must be made available to editors/reviewers. We strongly encourage code deposition in a community repository (e.g. GitHub). See the Nature Research guidelines for submitting code & software for further information.

Data

Policy information about availability of data

All manuscripts must include a data availability statement. This statement should provide the following information, where applicable:

- Accession codes, unique identifiers, or web links for publicly available datasets
- A list of figures that have associated raw data
- A description of any restrictions on data availability

Data are available upon request from the cohort authors with appropriate research agreements.

Field-specific reporting

Please select the one below that is the best fit for your research. If you are not sure, read the appropriate sections before making your selection.

☒ Life sciences ☐ Behavioural & social sciences ☐ Ecological, evolutionary & environmental sciences

For a reference copy of the document with all sections, see nature.com/documents/nr-reporting-summary-flat.pdf

Life sciences study design

All studies must disclose on these points even when the disclosure is negative.

Sample size	9,448
Data exclusions	n/a
Replication	Three independent cohorts for whole blood, one independent cohort using methylation from a different tissue, and one indepdendent cohort for assessment of replication when predicting another trait.
Randomization	All covariates are fit jointly to obtain partial regression coefficients. Age, sex, cell counts and PCs, alongside SNP marker data.
Blinding	analysts had no role in study collection or design, receiving only anonymous data

Reporting for specific materials, systems and methods

We require information from authors about some types of materials, experimental systems and methods used in many studies. Here, indicate whether each material, system or method listed is relevant to your study. If you are not sure if a list item applies to your research, read the appropriate section before selecting a response.

Materials & experimental systems

n/a	Involved in the study	
☒	☐	Antibodies
☒	☐	Eukaryotic cell lines
☒	☐	Palaeontology
☒	☐	Animals and other organisms
☐	☒	Human research participants
☒	☐	Clinical data

Methods

n/a	Involved in the study	
☒	☐	ChIP-seq
☒	☐	Flow cytometry
☒	☐	MRI-based neuroimaging

Human research participants

Policy information about studies involving human research participants

Population characteristics

Accessible Resource for Integrative Epigenomic Studies (ARIES, http://www.ariesepigenomics.org.uk/ and Relton et al 2015. Full details of the preprocessing and normalization of ARIES has been described previously Min et al 2018.

The TwinsUK registry consists of over 14,000 research volunteer twin participants from the United Kingdom who have joined since 1992, with equal numbers of same-sex monozygotic and dizygotic twin pairs who are predominately female (84\%). Data are collected through longitudinal questionnaires and clinical visits. The registry collects biological samples and further data collected through analysis of biological samples. DNA methylation profiles were generated using the Infinium HumanMethylation450 BeadChip in adipose tissue biopsies and whole blood samples from TwinsUK participants. Adipose tissue DNA methylation data were generated from subjects who were free from severe diseases, as previously described Grundberg et al. 2013. Ethical approval was granted by the National Research Ethics Service London-Westminster, the St Thomas' Hospital Research Ethics Committee (EC04/015 and 07/H0802/84). All research participants provided written informed consent prior to taking part in the study.

The Lothian Birth Cohort 1936 is a longitudinal study of aging and it follows 1,091 members of the 1947 Scottish Mental Survey, who were recontacted in later life, when they were living in the Edinburgh area of Scotland. The cohort members were all born in 1936 and have been assessed for a wide variety of health and lifestyle outcomes at ages 70, 73, 76, 79, and 82 years. DNA has been collected at each clinical visit. In the present study, we considered DNA methylation data (Illumina 450k array) from whole blood, taken at mean age 70, for analysis. Details of the collection and processing of the data have been reported previously McCartney et al. 2018.

Recruitment	See above statement and references included here and in full within the manuscript
Ethics oversight	provided above, in the acknowledgments and in the references provided.

Note that full information on the approval of the study protocol must also be provided in the manuscript.
