# Supplementary material for: Bayesian reassessment of the epigenetic architecture of complex traits
Source: Nat Commun. 2020 Jun 8;11:2865. doi: 10.1038/s41467-020-16520-1 (PMC7280277; doi:10.1038/s41467-020-16520-1)
Supplement: Supplementary file 1 — Supplementary Information [file 41467_2020_16520_MOESM1_ESM.pdf]

Editorial Note: This manuscript has been previously reviewed at another journal that is not operating a transparent peer review scheme. This document only contains reviewer comments and rebuttal letters for versions considered at *Nature Communications*. Mentions of prior referee reports have been redacted.

Reviewers' comments:

Reviewer #2 (Remarks to the Author):

I do not feel that the authors sufficiently responded to my earlier critiques, particularly relating to the much higher proportions of variance explained using this method than those previously reported.
1. Nature Communications does not have Methods papers, so I still think the results of the approach would be clearer if there were separate sections for the BMI and smoking applications. Right now, its still hard to follow jumping back and forth between the results.
2. I am not fully convinced by the author response explaining why there is more variance explained by their method than a previous effort in the same cohort. Using BMI as an example in this paper: https://genomebiology.biomedcentral.com/articles/10.1186/s13059-018-1514-1 , 1109 probes constituted a methylation score that explained ~12% of BMI's variance. In this current paper, all probes explain 66.74% of variance in BMI, with 144 probes explaining 65.5% of this variance. This would suggest that 144 probes explain ~43% of variance in BMI, which is 3 times higher than their previous paper using 8 times fewer probes or a 24 fold enrichment. For the proper interpretation of these data, the authors should run this approach fully adjusting for known confounders of epigenetic data to determine how much they are biasing these results, as their statement **[REDACTED]** actually worsens interpretability and usability since anyone actually implementing this approach on blood DNA methylation would adjust at least for cellular composition.
3. Response was adequate
4. Response was adequate

Reviewers' comments:

Reviewer #3 (Remarks to the Author):

The authors have addressed the previous comments. Two minor issues still remain. Page 3, line 70, text says 4 cell types, not 5. I think this should say 5, as the response to reviewer states. Please correct this in the paper. Regarding the previous comment about using a bold R, yes, residual was the wrong word. Please see Legend Fig S1 for 2 separate uses of a bold R.

Reviewer #4 (Remarks to the Author):

Reviewer 2

[REDACTED]

We thank Reviewer 2 for their comments, which have resulted in improved analyses that have shown a clearer picture of the usefulness of our method. We regret not addressing these in full in the first round of comments and we have now taken many steps to comprehensively address the concerns raised. We outline each of these in detail below.

[REDACTED]

We thank the reviewer for their comment. We agree that these findings are of great interest and we have now greatly expanded our results section and included a large number of additional analyses as follows:

- We have adapted our algorithm to fit potential confounders of sex, age, cell count composition, and the leading principal components of both the epigenetic and genetic data (lines 39-68, 162-164, Methods sec. 5.5.1. lines 508-525). When we fit these in the model alongside the methylation probes, our results did not change at all. This validates our simulation work, showing that unlike other approaches we do not need to fit PCs to control for potential confounding. It also validates our previous findings of a large effective number of epigenetic probe associations, spread throughout the genome, for BMI as opposed to a limited number for smoking behavior.
- We have doubled the sample size to 9,448 individuals, representing the largest methylation study to date. In this new data, our results remain the same, further validating our previous findings. We entirely replicate results of previous studies, as well as present novel findings. All probes with >95% inclusion probability for both BMI and smoking replicate previous findings, with the exception of three probes implicating two novel genes whose DNA methylation levels are associated with smoking behavior. This provides very strong support that our approach is appropriate and robust.
- In response to concerns below regarding the biological relevance of our findings we have extended our model to estimate probe associations, whilst taking into account genomic annotation information (lines 56-68, Figure 4, and lines 216-239). Most enrichment analyses, first run a standard analysis and then test whether associations are stronger in certain groups by "post hoc" testing. Our approach is unique as it estimates enrichment of the probe associations at the same time as estimating the associations themselves. For both traits we find genes with differential expression in whole blood are enriched in their effects as expected given the tissue. Other associations are then conditional on this. For BMI, we find methylation of genes that are differentially expressed in the adrenal gland, subcutaneous adipose, thyroid, fibroblasts showed larger effect sizes. For smoking, we find that probes mapping to genes that are differentially expressed in the aorta, the tibial artery, the brain spinal cord, and lymphocytes are those with larger probe effects. Associations for

BMI are spread across tissue-specific genes more so than for smoking highlighting the large effective number of BMI associations in blood spread across biological processes. These are all novel findings and this demonstrates that our associations reflect associations with underlying biological pathways.

- We use our results to predict BMI in three independent co-horts from methylation in whole blood, adding an additional cohort from our previous manuscript (lines 240-279 and Table 1). The amount of phenotypic variation explained by any predictor (whether a polygenic risk score, methylation predictor, etc.) within an independent sample has an expectation from theory (for eample see this Nature Reviews Genetics paper: PMCID: PMC4096801 DOI: 10.1038/nrg3457). Our results are exactly in-line with these expectations – given 76% variance captured in the initial data set, we would expect 25% variance explained by the predictor in an independent cohort. Obtaining values that match theory is evidence that the results we obtain do not simply reflect cell-type or experimental confounding but rather real associations that replicate across data sets.
- This expectation relies on associations being the same across ages. We provide the first evidence for age-specific effects for BMI. In a regression of the phenotype on the predictor, the regression slope should be 1 (i.e. the predictor and the phenotype one is trying to predict should be on the same scale). If it is less than 1, then it implies that the predictor is not on the same scale as the phenotype and thus that the trait-methylation associations of the original data set do not have the same relevance (i.e. do not have a correlation of one) with those in the prediction data. Here, we find that in an age matched sample (TwinsUK) we achieve prediction accuracy of 30.8% with a slope slightly less than 1, again validating our results that large amount of variation is captured in our original data set and that the effects are meaningful as they translate to high prediction accuracy in an independent cohort. For younger cohorts (ARIES) or older cohorts (LBC), slopes and prediction accuracies were lower, which is expected with age-specific trait-methylation associations that are a phenotypic consequence rather than a cause, further supporting our previous results.
- We also replicate our results across tissues. There are no large-scale publicly available tissue-specific methylation data sets with BMI measures. Thus, we have collaborated with King's College London, who have the second largest methylation study in both whole-blood and adipose tissue. We obtain 9% prediction accuracy when using whole blood trait-methylation estimates to predict the BMI-methylation relationship in adipose tissue. This further confirms that our results are not due to confounding in whole blood, but rather reflects meaningful associations. The slope was 0.4, which is an estimate of the correlation of methylation associations from whole blood to adipose tissue. The sample size of the adipose data set is <400, meaning that the 95% credible intervals on any direct estimate of the variance explained would span 0 to 0.8 making this comparison ineffective. Instead, we focus on prediction accuracy achieved using our whole blood results and our estimate of 9% adjusted r-squared in an independent sample, with a starting sample size of 9,500 individuals and 20,000 independent genes, gives an expectation that 50% of phenotypic variation should be captured by methylation probes in adipose tissue.
- We also assessed the variation captured by our predictors for other traits finding in LBC wave one that our smoking predictor captures 7.2% of the variance for forced expiratory volume, and our BMI predictor captures 11.2% of the variance of triglyceride levels in blood and 7.6% of the variance of high density lipoprotein levels in blood. These results, along with our enrichment analyses, further show that our approach captures a signal related to the relevant biological processes underlying these phenotypes.
- Finally, we also compared our estimates against previously published effect sizes. We find that they are comparable in scale and sign for the bigger effects (those which are more

identifiable). Our model then shrinks smaller effect sizes toward zero where there is less power to identify them. This demonstrates that our associations map to previous results (all of the BMI probes with >95% IP replicate previous findings), and also shows that previous results are likely to be inflated due to reporting bias. When trait-probe associations are estimated jointly the effect sizes decline in a consistent manner further demonstrating the advantage of our approach. We have contextualizing the effect sizes detected with previous results from different tissues figure 3 panels e and f.

This has resulted in an expanded and vastly more comprehensive analysis of these two phenotypes, highlighting the improvements in inference that can be gained from our approach. All of this evidence supports our conclusions that our learned models are unaffected by confounders and its predictive ability is related to the traits and not to external confounders. It is clear that our results reflect meaningful trait-methylation associations as: (1) they are consistent with increased sample size; (2) they are consistent irrespective of whether known potential confounders are adjusted for in the analysis or not; (3) the effects we obtain cluster in biological pathways association with the phenotypes; (4) prediction accuracy is high and entirely consistent with theory; (5) prediction accuracy decays with samples of younger and older ages which would not happen if results reflected confounding; (6) results replicate from whole blood to adipose tissue with a prediction accuracy that implies a large amount of phenotypic variation captured by methylation probes; and (7) our predictor can predict correlated phenotypes, which again would not be the case if our results simply reflected confounding.

We would also like to highlight that the model and the implementation is entirely novel and thus while the findings we present are of interest, this is also a methods paper and it is very important to formally benchmark our approach against others in the field. In the expanded results section, we chose to keep smoking and BMI results together, given that they complement each other and contrast each other in key areas, BMI having lots of small effects and smoking have fewer but bigger effects. We think that in this format the reader can better distinguish how the two traits differ from each other while from the summaries we produced.

[REDACTED]

We thank the reviewer for their comments here. However, there appears to be a very important misunderstanding. Referring to this paper: https://genomebiology.biomedcentral.com/articles/10.1186/s13059-018-1514-1 the reviewer states:

[REDACTED]

This statement is not correct. The 12% value referred to is the prediction accuracy obtained in an independent cohort and not an estimate of the variation explained in the original data. This 12% estimate should be compared to our estimate of 19.5% in the same LBC cohort (highlighting the improvement of our approach for prediction accuracy), it should not be compared to the 75.7% as explained above and in the literature - i.e. Nature Reviews Genetics paper: PMCID: PMC4096801 DOI: 10.1038/nrg3457 contains many references). As we explain above an estimate of 19.5% prediction accuracy is entirely in-line with the estimates of the variation explained in the original training data.

Nevertheless, as outlined above, we have adapted our algorithm to fit potential confounders of sex, age, cell count composition, and the leading principal components of both the epigenetic and genetic data. When we fit these in the model alongside the methylation probes, our results did not change at all. This validates our simulation work, showing that unlike other approaches we do not need to fit PCs to control for potential confounding. It also validates our previous findings of a large effective number of epigenetic probe associations, spread throughout the genome, for BMI as opposed to a limited number for smoking behavior. As we also wish to take a cautious approach, we have highlighted in the discussion that separating correlated effects is a difficult thing to do and that we can only draw inference from what we have observed (lines 304 -323), however, we believe that this inference is robust to potential confounding.

[REDACTED]

We thank the reviewer for their comment here. As there are no large-scale comparable publicly available data sets with adipose tissue or lung tissue, we collaborated with Dr. Jordana Bell's laboratory at King's College London, who have the second largest methylation study in both whole-blood and adipose tissue. We obtain 9% prediction accuracy when using whole blood trait-methylation estimates to predict the BMI-methylation relationship in adipose tissue. This further confirms that our results are not due to confounding in whole blood, but rather reflect meaningful associations. The slope was 0.4, which is an estimate of the correlation of methylation associations from whole blood to adipose tissue. The sample size of the adipose data set is <400, meaning that the 95% credible intervals on any direct estimate of the variance explained would span 0 to 0.8 making this comparison ineffective. Instead, we focus on prediction accuracy achieved using our whole blood results and our estimate of 9% adjusted r-squared in an independent sample, with a starting sample size of 9,500 individuals and 20,000 independent genes, gives an expectation that 50% of phenotypic variation should be captured by methylation probes in adipose tissue. This really is the best that we can do given current data. Our approach is best suited to data sets in the thousands, rather than the hundreds, which are being generated and will likely be available in future.

We also took the following steps regarding the biological relevance of our findings:

- We have extended our model to estimate probe associations, whilst taking into account genomic annotation information (lines 56-68, Figure 4, and lines 216-239). Most enrichment analyses, first run a standard analysis and then test whether associations are stronger in certain groups by "post hoc" testing. Our approach is unique as it estimates enrichment of the probe associations at the same time as estimating the associations themselves. For both traits we find genes with differential expression in whole blood are enriched in their effects as expected given the tissue. Other associations are then conditional on this. For

BMI, we find methylation of genes that are differentially expressed in the adrenal gland, subcutaneous adipose, thyroid, fibroblasts showed larger effect sizes. For smoking, we find that probes mapping to genes that are differentially expressed in the aorta, the tibial artery, the brain spinal cord, and lymphocytes are those with larger probe effects. Associations for BMI are spread across tissue-specific genes more so than for smoking highlighting the large effective number of BMI associations in blood spread across biological processes. These are all novel findings and this demonstrates that our associations reflect associations with underlying biological pathways.

- We provide the first evidence for age-specific effects for BMI (Table 1). In a regression of the phenotype on the predictor, the regression slope should be 1 (i.e. the predictor and the phenotype one is trying to predict should be on the same scale). If it is less than 1, then it implies that the predictor is not on the same scale as the phenotype and thus that the trait-methylation associations of the original data set do not have the same relevance (i.e. do not have a correlation of one) with those in the prediction data. Here, we find that in an age matched sample (TwinsUK) we achieve prediction accuracy of 30.8% with a slope slightly less than 1, again validating our results that large amount of variation is captured in our original data set and that the effects are meaningful as they translate to high prediction accuracy in an independent cohort. For younger cohorts (ARIES) or older cohorts (LBC), slopes and prediction accuracies were lower, which is expected with age-specific trait-methylation associations that are a phenotypic consequence rather than a cause, further supporting our previous results as we would not observe this pattern if our results simply captured confounding.
- We performed GO ontology enrichment analyses over the new set of probes found with 95% IP , lines 177-181 and updated tables S6 and S7.
- We also assessed the variation captured by our predictors for other traits finding in LBC wave one that our smoking predictor captures 7.2% of the variance for forced expiratory volume, and our BMI predictor captures 11.2% of the variance of triglyceride levels in blood and 7.6% of the variance of high density lipoprotein levels in blood (lines 261-266). These results, along with our enrichment analyses, further show that our approach captures a signal related to the relevant biological processes underlying these phenotypes. As confounding is unlikely to be similar across traits, we feel this is further evidence that our results are biologically meaningful.

We appreciate this point raised by Reviewer 2 and we regret not demonstrating this fully in the previous version of our manuscript. We hope our efforts to address the points raised are appreciated and we feel that this manuscript now represents the most comprehensive trait-methylation association study conducted to date, using one of the most sophisticated analysis techniques, in the largest sample size currently available.

[REDACTED]

We thank the reviewer for their excellent suggestion here. We compared our estimates against previously published effect sizes from the EWAS catalogue. Our associations map to previous results as all of the BMI probes with >95% IP replicate previous findings and are congruent in magnitude and directionality (Figure 3). These are the larger effects, which are more identifiable, and our model, which estimates trait-probe associations jointly, then 'shrinks down' smaller effects into a small variance group where there is less power to identify them and thus the effect sizes decline in a consistent manner. When we compare these smaller effects to those reported in the literature, we find that previous results are likely to be inflated due to reporting bias. This is because effect sizes estimated without regularization will tend to be systematically over-estimated as power decreases (because estimation error increases). This over-estimation is magnified if only significant effects are reported as in the EWAS catalogue. This demonstrates the advantage of our approach. A large number of CPG sites that we detect as small effects do replicate result of previous studies, but we determine their effects to be much smaller than previously reported when they are estimated conditionally on the other large effect probes within our model. This is not an artifact of our model, rather we are correctly estimating the small effects sizes and this is pivotal as we show that including these effect in the model and thus into a predictor improves accuracy in out of sample prediction. Our method while helping us identify the most significant probes, does not discard possible sources of information in the form of distribution over smaller effects, effects that would be both mis-estimated and/or discarded without a regularized regression framework. Additionally, by working in a Bayesian framework we quantify the uncertainty caused by low power over these estimates. Thus, as the reviewer correctly points out, BMI does not appear to have strong epigenetic effects, and we can recover the weak epigenetic effects in our framework, with their corresponding uncertainty in the form of posterior inclusion probabilities.

We have now updated figure 3, panels a,b,c and d present the new set of probes with 95%along with their presence in the EWAS catalog. Panels e and f show the comparisons between our effect sizes and those in the EWAS catalog, were we can see clearly how the congruency between both sources diminishes as we diminish the PIP.

We additionally show that our estimates are a) unchanged if we adjust for confounders, b) the predictive power of the PCs of the most significant probes is independent to their correlation with the cell counts; BMI PCs have low predictive power and higher correlation to the cell counts while for smoking the PCs have high predictive power but weak correlation with the cell counts (lines 510-527). Taking the PCs of these probes and regress over phenotypes only tell us if there are common axes of variation among these probes that are associated with the phenotype, it would not help us elucidate the nature of their explanatory power as well as we have done by proving points a) and b).

Reviewer 3

The authors have addressed the previous comments. Two minor issues still remain. Page 3, line 70, text says 4 cell types, not 5. I think this should say 5, as the response to reviewer states. Please correct this in the paper. Regarding the previous comment about using a bold R, yes, residual was the wrong word. Please see Legend Fig S1 for 2 separate uses of a bold R.

We thank the reviewer for their remaining concerns and for their previous efforts. We have now addressed all of these remaining issues on line 70 and in the legends of Fig S1 and S2.

REVIEWERS' COMMENTS:

Reviewer #2 (Remarks to the Author):

The authors addressed my concerns

Detailed point-by-point response

REVIEWER #2 (Remarks to the Author):
The authors addressed my concerns

We thank Reviewer #2 for their previous concerns and their contributions in improving the manuscript. We are very pleased to have addressed the concerns raised.
