# Supplementary material for: Bayesian reassessment of the epigenetic architecture of complex traits
Source: Nat Commun. 2020 Jun 8;11:2865. doi: 10.1038/s41467-020-16520-1 (PMC7280277; doi:10.1038/s41467-020-16520-1)
Supplement: Supplementary file 2 — Peer Review File [file 41467_2020_16520_MOESM2_ESM.pdf]

Supplementary Information

Bayesian reassessment of the epigenetic architecture of complex traits

Trejo Banos et al.

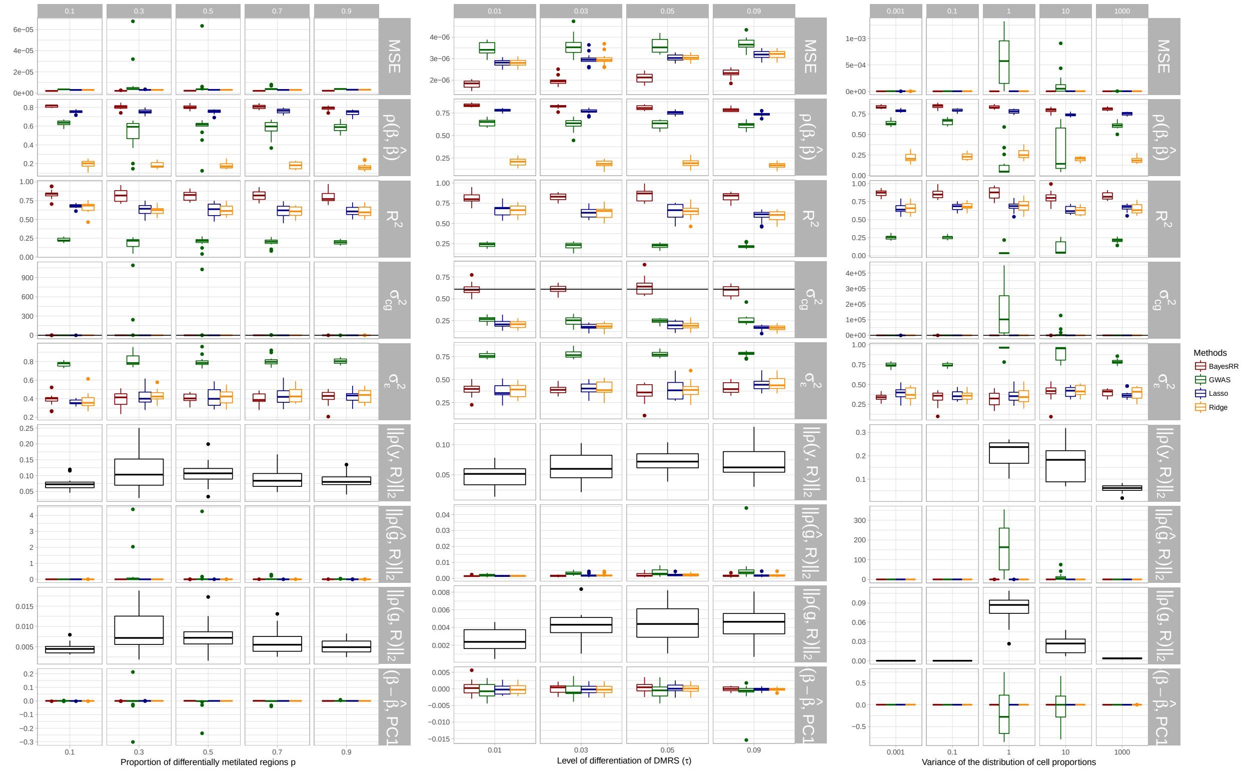

Supplementary Figure 1. Simulation results of multi-probe approaches without latent factor correction with single-probe least squares regression included as a baseline. Boxplots of distribution of scores, the line in the middle of the box represents the median, upper and lower bounds of the box represent first and third quartiles respectively, whiskers represent datum up to 1.5 interquartile distance from box bounds. Estimation of phenotype-epigenetic associations comparing BayesRR to other multi-probe association methods that do not include latent factors within the model (LASSO and Ridge regression as implemented in glmnet), with a baseline of single-probe least squares regression (GWAS, see Methods), across different proportions of differentially methylated regions (DMRS), different levels of differentiation of of the DMRS, and different variances of the cell-type proportions. In many of these scenarios, probes are associated with cell-type proportion variation and the degree of cell-type proportion confounding is given by the norm of the correlation vector between the phenotype and the cell-type proportions that is shown in row panel ($||\rho(\mathbf{R}, \mathbf{y})||$). The remaining panels give the mean square error (MSE), the correlation between true effects and estimates ($\rho(\beta, \hat{\beta})$), the variance attributable to the top probes ($\mathbf{R}^2$), the phenotypic variance attributable to the probes (σ^2_{cg}), the error variance (σ^2_e), the norm of the correlation vector between a individual-level predictor made from the probe effects and the cell-type proportions ($||\rho(\mathbf{R}, \hat{\mathbf{g}})||$), the norm of the correlation vector between the true individual-level genetic value and the cell-type proportions ($||\rho(\mathbf{R}, \mathbf{g})||$), and the correlation between the first principal component of the probe data and the difference between the estimated and true effect ($||\rho(\mathbf{P}, \mathbf{R})||$). Across all simulation settings and degrees of cell-type confounding, BayesRR outperforms comparable multi-probe association methods and returns associations that are unbiased of cell-type effects.

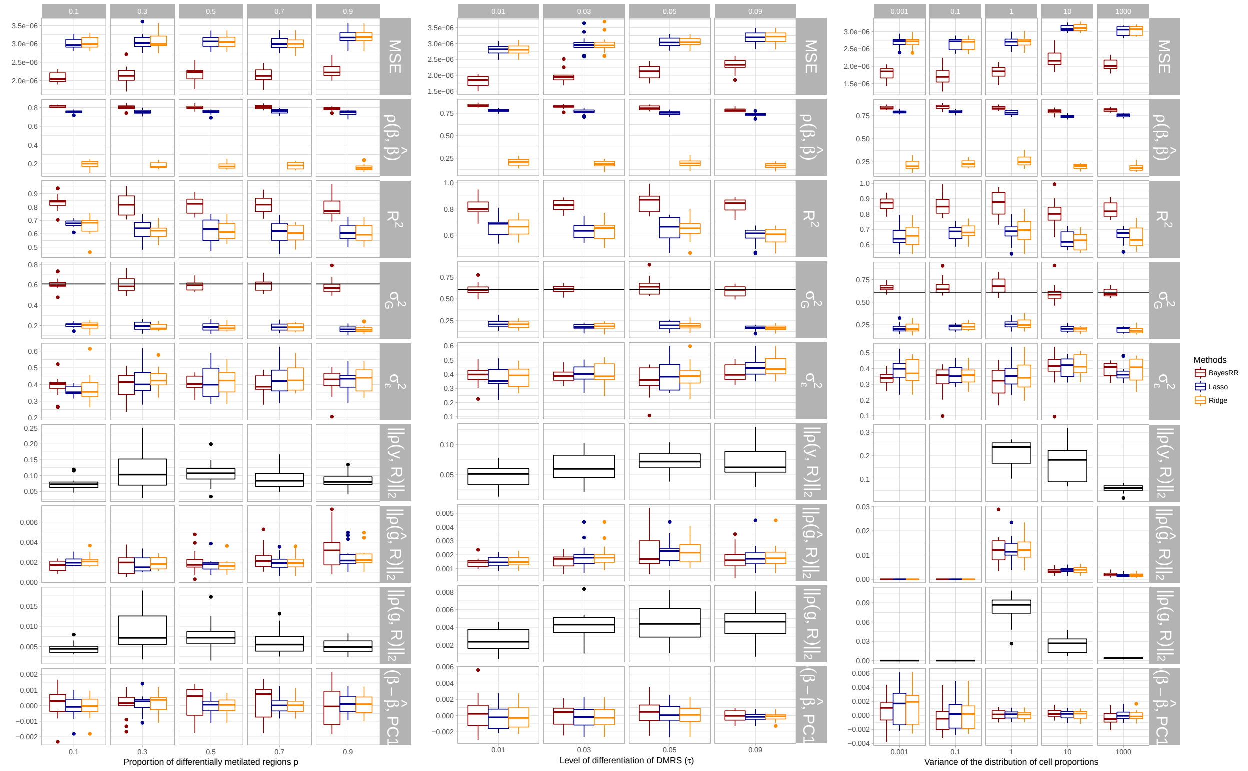

Supplementary Figure 2. Simulation results of multi-probe approaches without latent factor correction. Boxplots of distribution of scores, the line in the middle of the box represents the median, upper and lower bounds of the box represent first and third quartiles respectively, whiskers represent datum up to 1.5 interquartile distance from box bounds. Estimation of phenotype-epigenetic associations comparing BayesRR to other multi-probe association methods that do not include latent factors within the model (LASSO and Ridge regression as implemented in glmnet, see Methods), across different proportions of differentially methylated regions (DMRS), different levels of differentiation of of the DMRS, and different variances of the cell-type proportions. In many of these scenarios, probes are associated with cell-type proportion variation and the degree of cell-type proportion confounding is given by the norm of the correlation vector between the phenotype and the cell-type proportions that is shown in row panel ($||\rho(\mathbf{R}, \mathbf{y})||$). The remaining panels give the mean square error (MSE), the correlation between true effects and estimates ($\rho(\beta, \hat{\beta})$), the variance attributable to the top probes ($\mathbf{R}^2$), the phenotypic variance attributable to the probes (σ^2_{cg}), the error variance (σ^2_e), the norm of the correlation vector between a individual-level predictor made from the probe effects and the cell-type proportions ($||\rho(\mathbf{R}, \hat{\mathbf{g}})||$), the norm of the correlation vector between the true individual-level genetic value and the cell-type proportions ($||\rho(\mathbf{R}, \mathbf{g})||$), and the correlation between the first principal component of the probe data and the difference between the estimated and true effect ($||\rho(\mathbf{P}, \mathbf{R})||$). Across all simulation settings and degrees of cell-type confounding, BayesRR outperforms comparable multi-probe association methods and returns associations that are unbiased of cell-type effects.

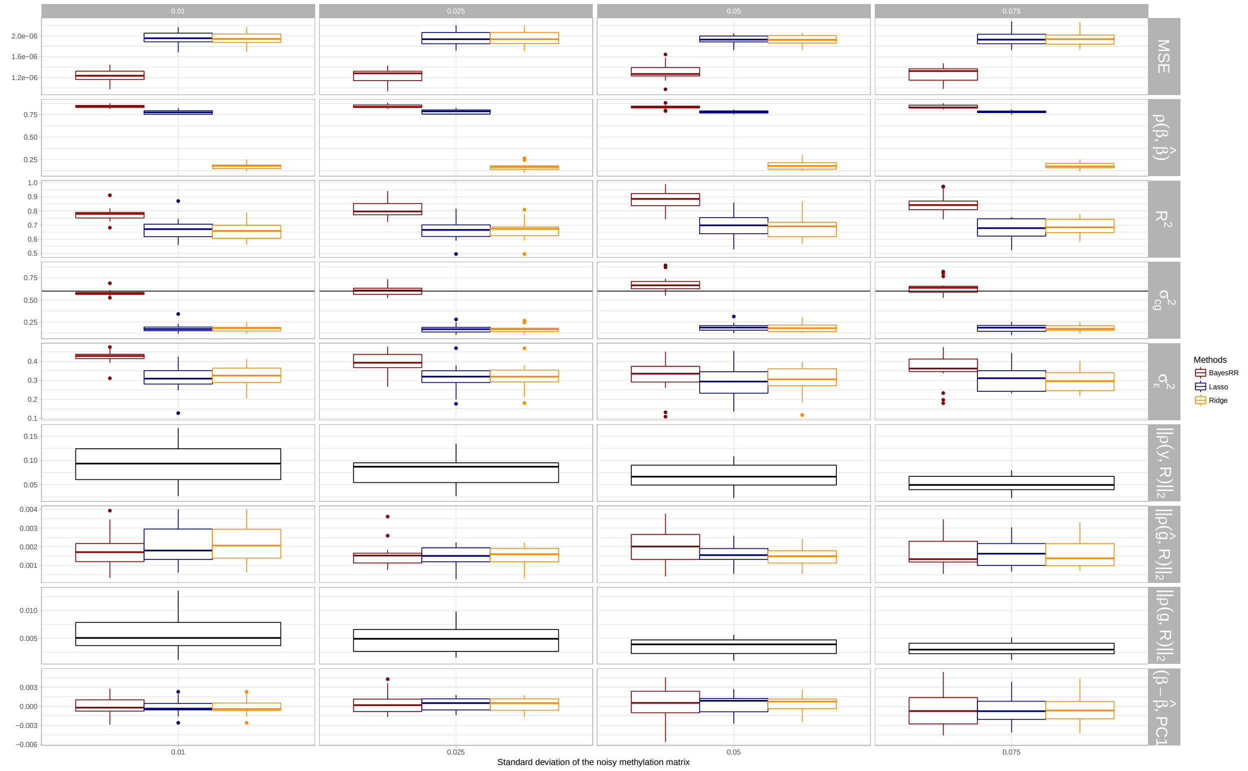

Supplementary Figure 3. Simulation results of multi-probe approaches without latent factor correction with varying degrees of noise added to the probe data. Estimation of phenotype-epigenetic associations comparing BayesRR to other multi-probe association methods that do not include latent factors within the model (LASSO and Ridge regression as implemented in glmnet, see Methods), across different SD of random noise added to the methylation data. In many of these scenarios, probes are associated with cell-type proportion variation and the degree of cell-type proportion confounding is given by the norm of the correlation vector between the phenotype and the cell-type proportions that is shown in row panel ($||\rho(\mathbf{R}, \mathbf{y})||$). The remaining panels give the mean square error (MSE), the correlation between true effects and estimates ($\rho(\beta, \hat{\beta})$), the variance attributable to the top probes ($\mathbf{R}^2$), the phenotypic variance attributable to the probes (σ^2_{cg}), the error variance (σ^2_e), the norm of the correlation vector between a individual-level predictor made from the probe effects and the cell-type proportions ($||\rho(\mathbf{R}, \hat{\mathbf{g}})||$), the norm of the correlation vector between the true individual-level genetic value and the cell-type proportions ($||\rho(\mathbf{R}, \mathbf{g})||$), and the correlation between the first principal component of the probe data and the difference between the estimated and true effect ($||\rho(\mathbf{P}, \mathbf{R})||$). Even with increased degrees of experimental and measurement error, BayesRR outperforms comparable multi-probe association methods.

a

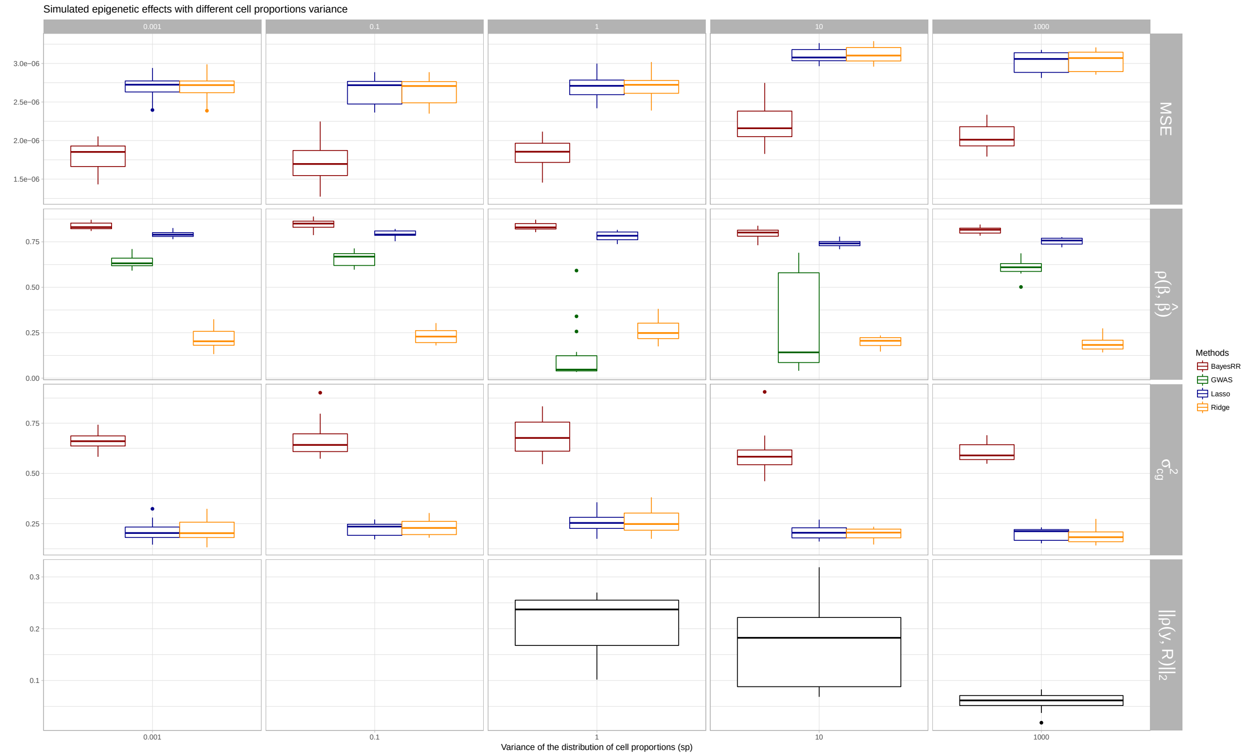

Supplementary Figure 4. Simulation results of multi-probe approaches without latent factor correction with single-probe least squares regression included as a baseline for model that jointly estimated both SNP marker and methylation probe associations. Boxplots of distribution of scores, the line in the middle of the box represents the median, upper and lower bounds of the box represent first and third quartiles respectively, whiskers represent datum up to 1.5 interquartile distance from box bounds. Estimation of phenotype-epigenetic associations comparing BayesRR to other multi-probe association methods that do not include latent factors within the model (LASSO and Ridge regression as implemented in glmnet), with a baseline of single-probe least squares regression (GWAS, see Methods), across different variances of the cell-type proportions. Results are shown for the methylation probe associations only as this is the focus of this work and we wished to assess whether the performance differed when another set of correlated covariates were in the model. In many of these scenarios, probes are associated with cell-type proportion variation and the degree of cell-type proportion confounding is given by the norm of the correlation vector between the phenotype and the cell-type proportions that is shown in row panel ($||\rho(\mathbf{R}, \mathbf{y})||$). The remaining panels give the mean square error (MSE), the correlation between true effects and estimates ($\rho(\beta, \hat{\beta})$), the variance attributable to the top probes ($\mathbf{R}^2$), the phenotypic variance attributable to the probes (σ^2_{cg}), the error variance (σ^2_e), the norm of the correlation vector between a individual-level predictor made from the probe effects and the cell-type proportions ($||\rho(\mathbf{R}, \hat{\mathbf{g}})||$), the norm of the correlation vector between the true individual-level genetic value and the cell-type proportions ($||\rho(\mathbf{R}, \mathbf{g})||$), and the correlation between the first principal component of the probe data and the difference between the estimated and true effect ($||\rho(\mathbf{P}, \mathbf{R})||$). Across all simulation settings and degrees of cell-type confounding, BayesRR outperforms comparable multi-probe association methods and returns associations that are unbiased of cell-type effects and unbiased of the SNP marker effects.

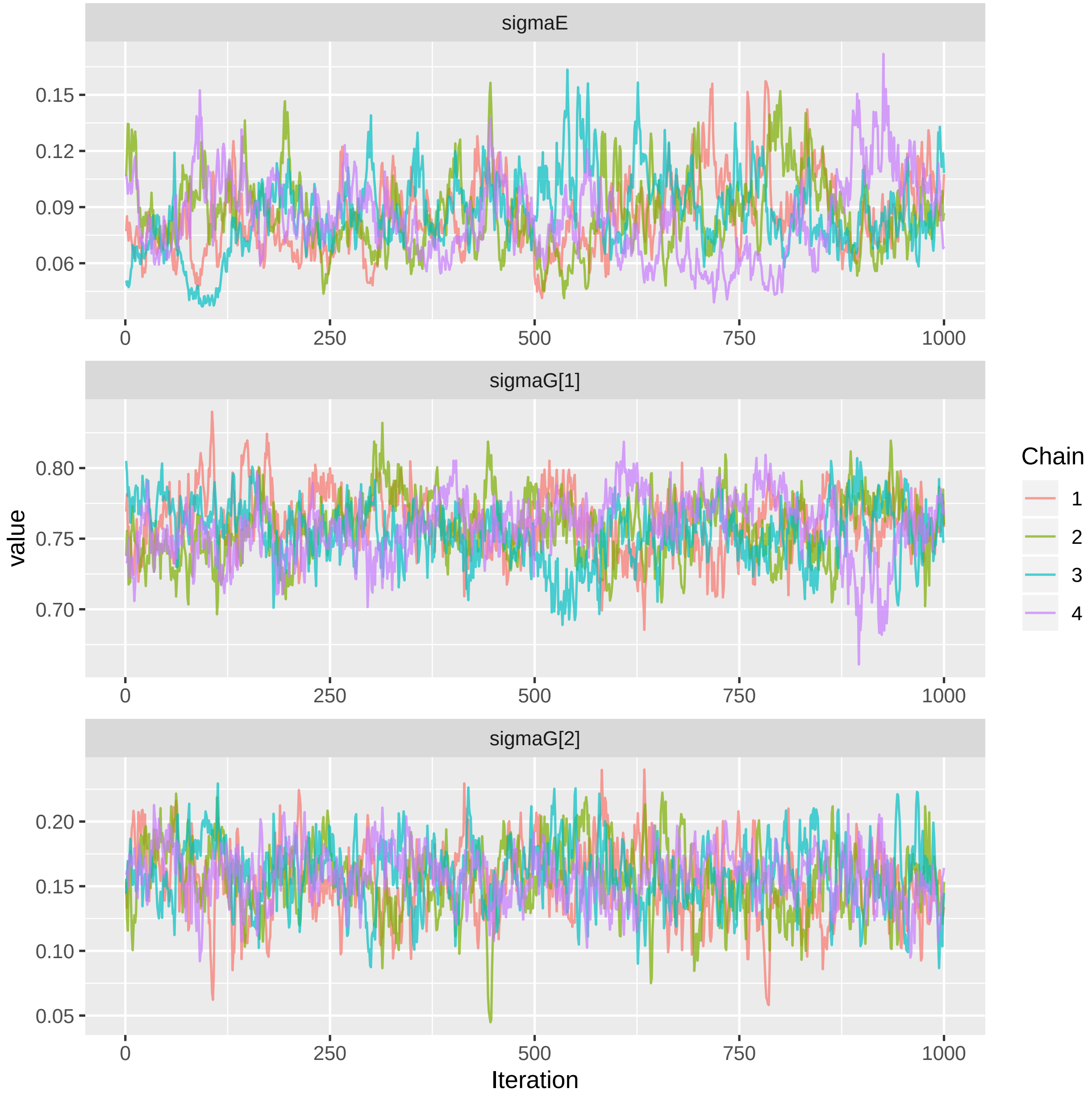

Supplementary Figure 5. Traceplot for the hyper-parameters of the body mass index (BMI) model in the Generation Scotland dataset. Four chains for BMI were run for 20000 samples with a burn-in of 10000 samples and thinning of 10 samples. Row panels give the estimates for the error variance $sigmaE(\sigma_\epsilon^2)$, the variance attributable to SNP marker effects $sigmaG[2](\sigma_G^2)$, and the phenotypic variance associated with methylation probes $sigmaG[1](\sigma_\phi^2)$.

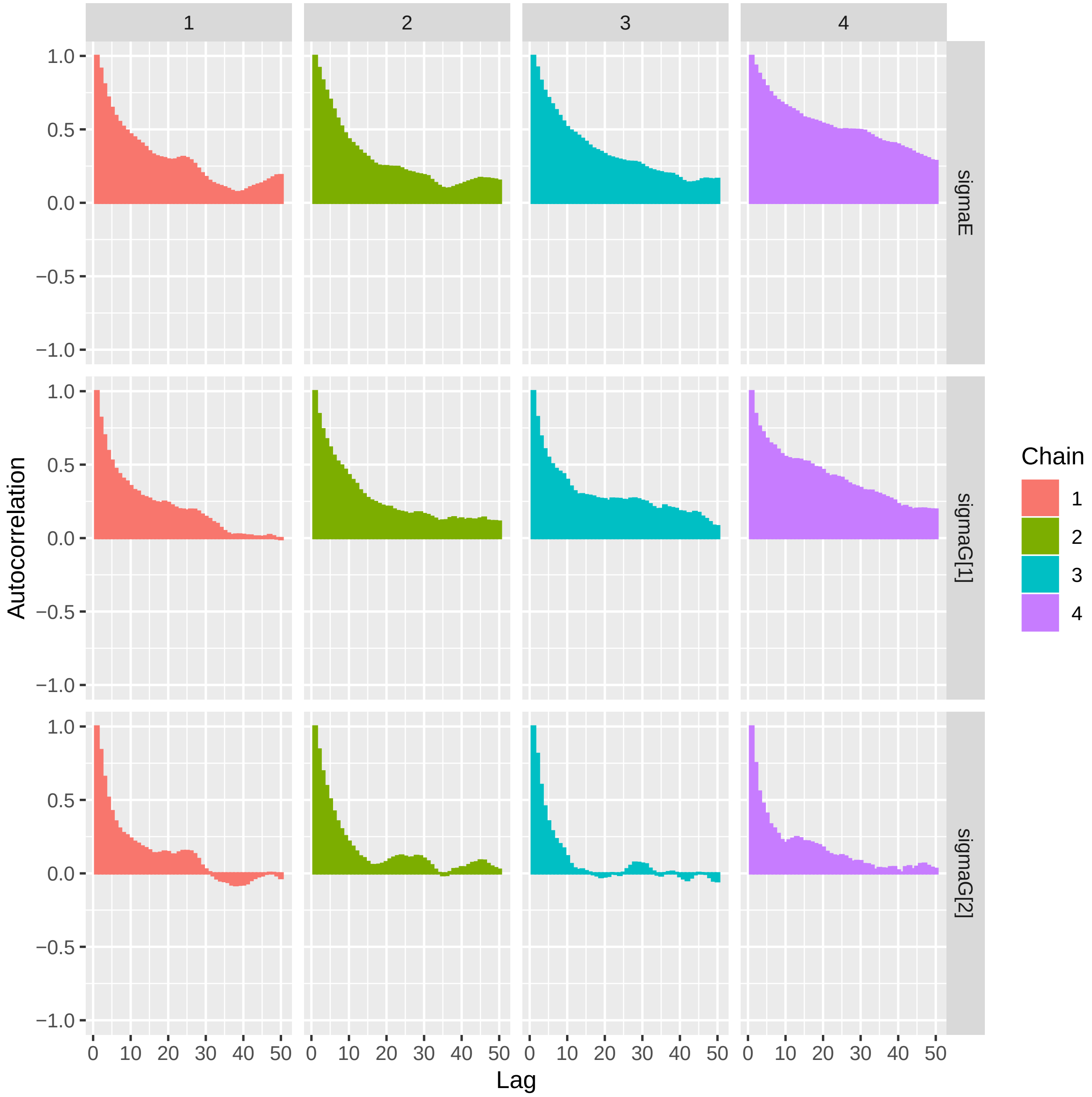

Supplementary Figure 6. Autocorrelation plot for the hyper-parameters of the body mass index (BMI) model in the Generation Scotland dataset. Four chains for BMI were run for 20000 samples with a burn-in of 10000 samples and thinning of 10 samples. Row panels give the autocorrelation for the error variance $sigmaE(\sigma^2_\epsilon)$, the variance attributable to SNP marker effects $sigmaG[2](\sigma^2_G)$, and the phenotypic variance associated with methylation probes $sigmaG[1](\sigma^2_\phi)$.

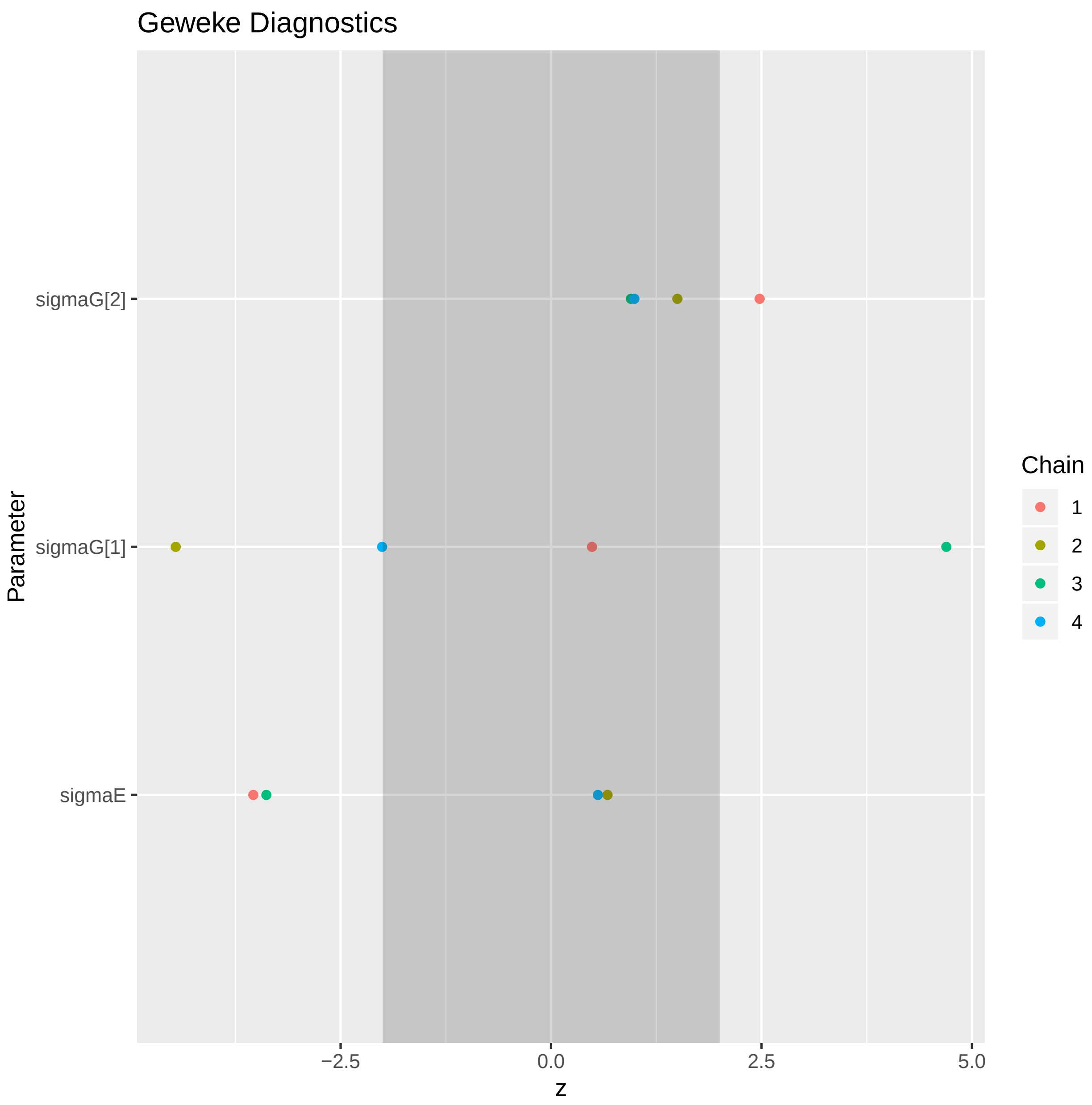

Supplementary Figure 7. Geweke z-score diagnostic for the hyper-parameters of the body mass index (BMI) model in the Generation Scotland dataset. Results for the four chains for the estimates of the the error variance $sigmaE(\sigma^2_\epsilon)$, the variance attributable to SNP marker effects $sigmaG[2](\sigma^2_G)$, and the phenotypic variance associated with methylation probes $sigmaG[1](\sigma^2_\phi)$, were compared using the Geweke z-score diagnostic with the acceptance interval(-2,2) highlighted in dark gray. Most chains reached the same parameter estimates, although there is some evidence of chain-level variance, meaning some effect of the starting values.

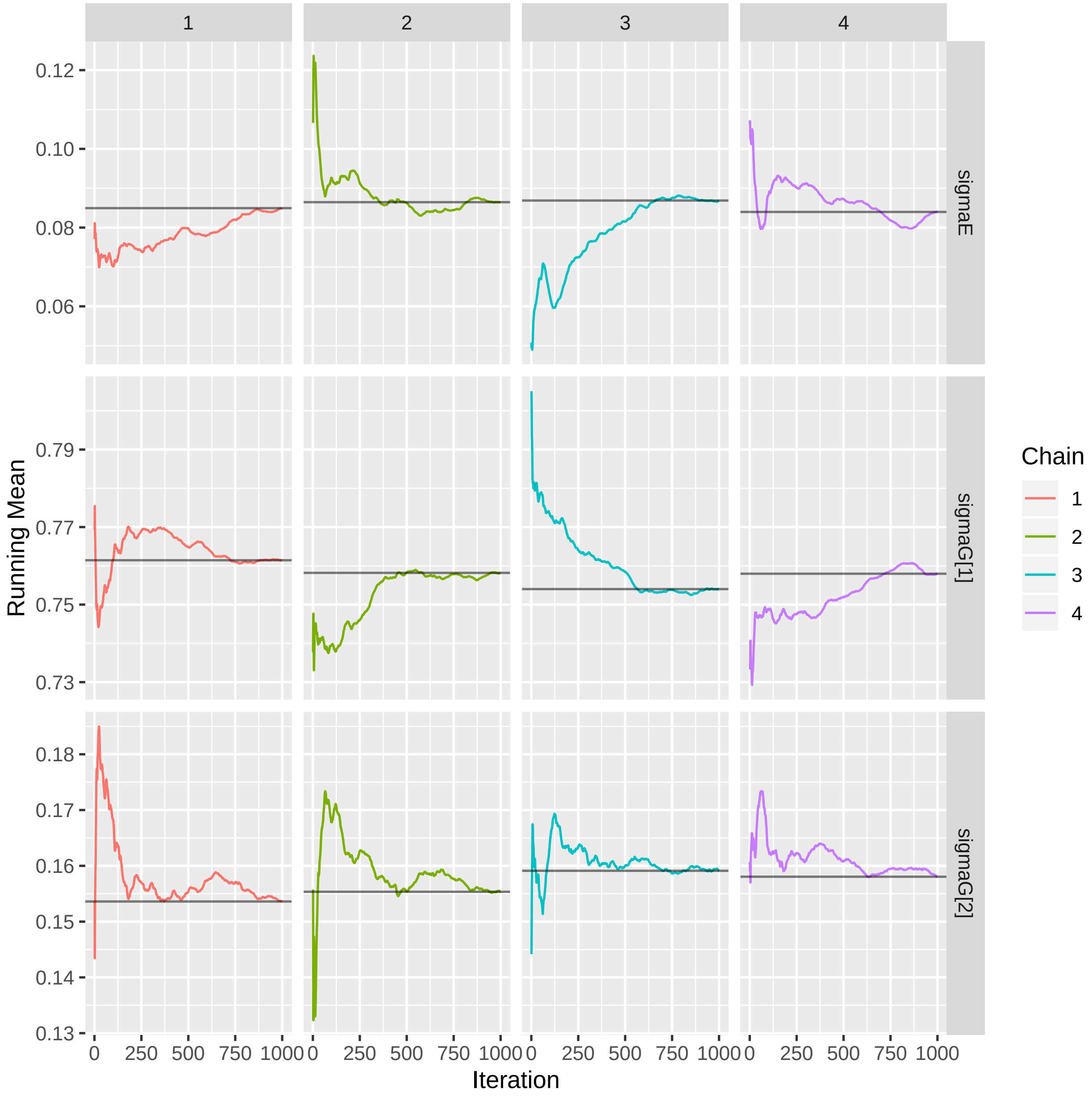

Supplementary Figure 8. Running means plot for the hyper-parameters of the body mass index (BMI) model in the Generation Scotland dataset. The mean of the chain at each sample is compared against the chains mean(horizontal line) for the estimates of tthe error variance $sigmaE(\sigma_{\epsilon}^2)$, the variance attributable to SNP marker effects $sigmaG[2](\sigma_G^2)$, and the phenotypic variance associated with methylation probes $sigmaG[1](\sigma_{\phi}^2)$. Given this, and the evidence of the among chain variance we combined the chains were thinned further to achieve 100 samples per chain.

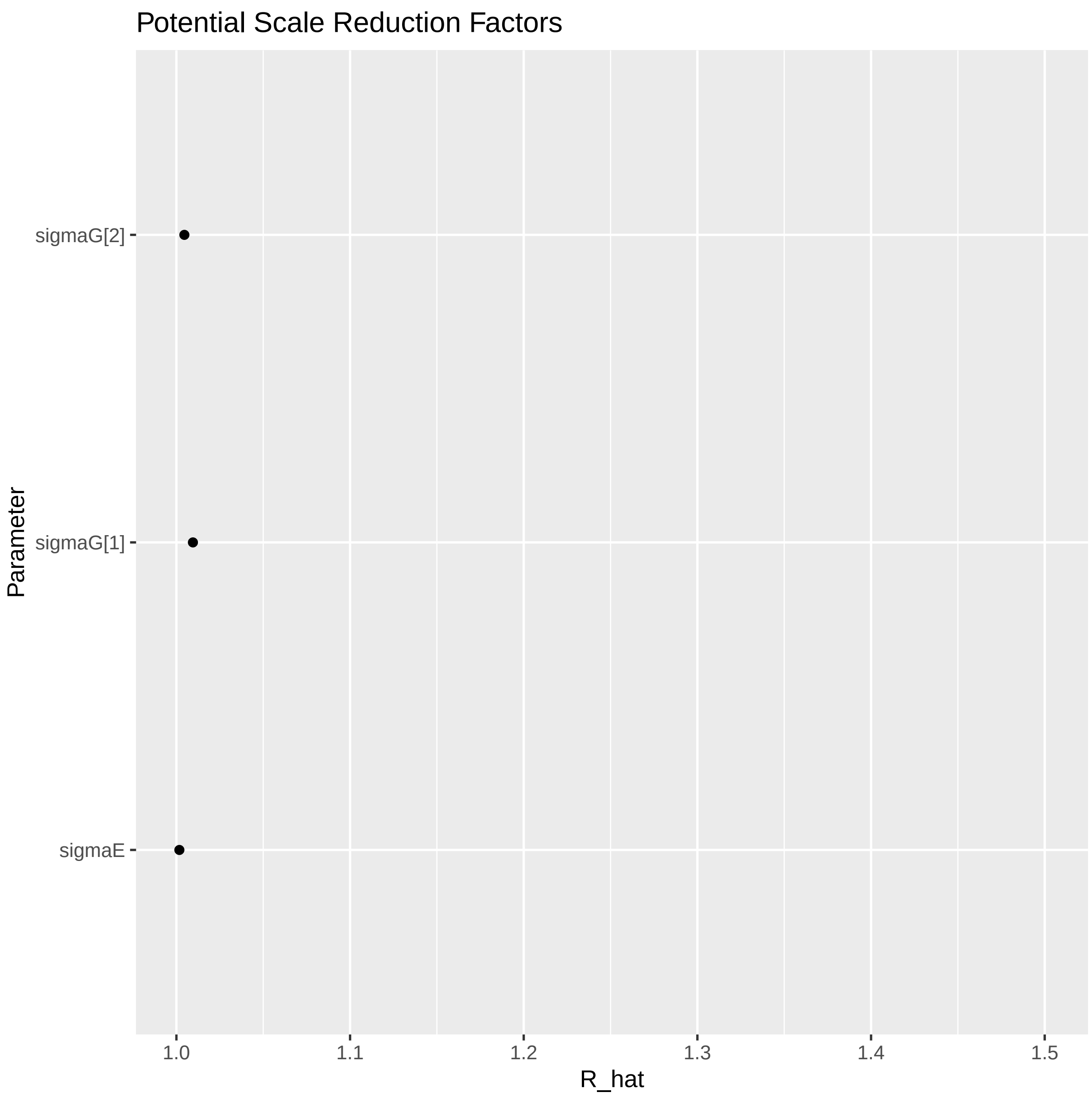

Supplementary Figure 9. Gelman R_{hat} diagnostic for the hyper-parameters of the body mass index (BMI) model in the Generation Scotland dataset. Results for the four chains for the estimates of the the error variance $sigmaE(\sigma_{\epsilon}^2)$, the variance attributable to SNP marker effects $sigmaG[2](\sigma_G^2)$, and the phenotypic variance associated with methylation probes $sigmaG[1](\sigma_{\phi}^2)$, were compared using the R_{hat} diagnostic, values lower than 1.1 are suggestive of convergence between chains.

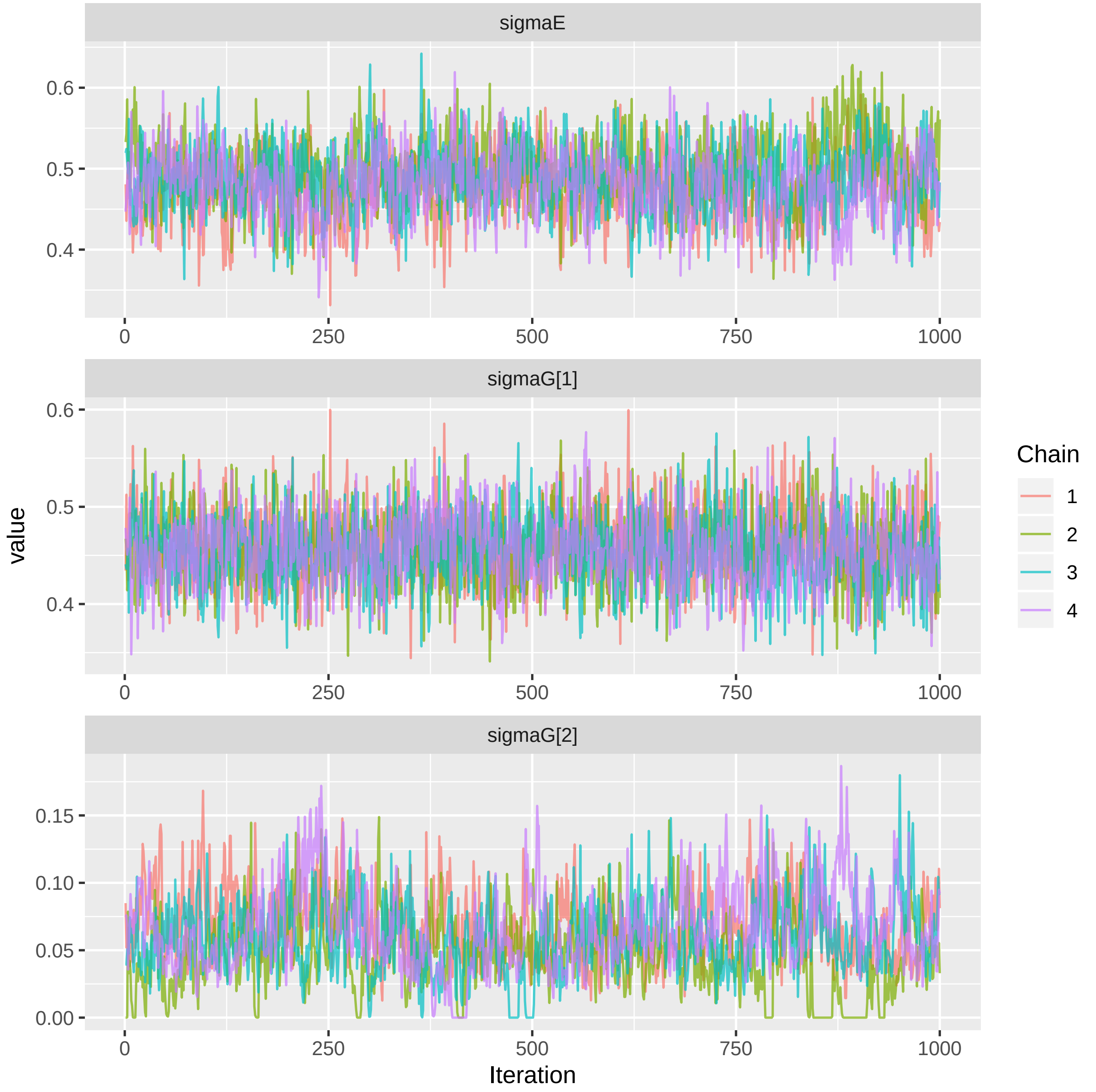

Supplementary Figure 10. Traceplot for the hyper-parameters of the smoking model in the Generation Scotland dataset. Four chains for smoking were run for 20000 samples with a burn-in of 10000 samples and thinning of 10 samples. Row panels give the estimates for the error variance $sigmaE(\sigma_\epsilon^2)$, the variance attributable to SNP marker effects $sigmaG[2](\sigma_G^2)$, and the phenotypic variance associated with methylation probes $sigmaG[1](\sigma_\phi^2)$.

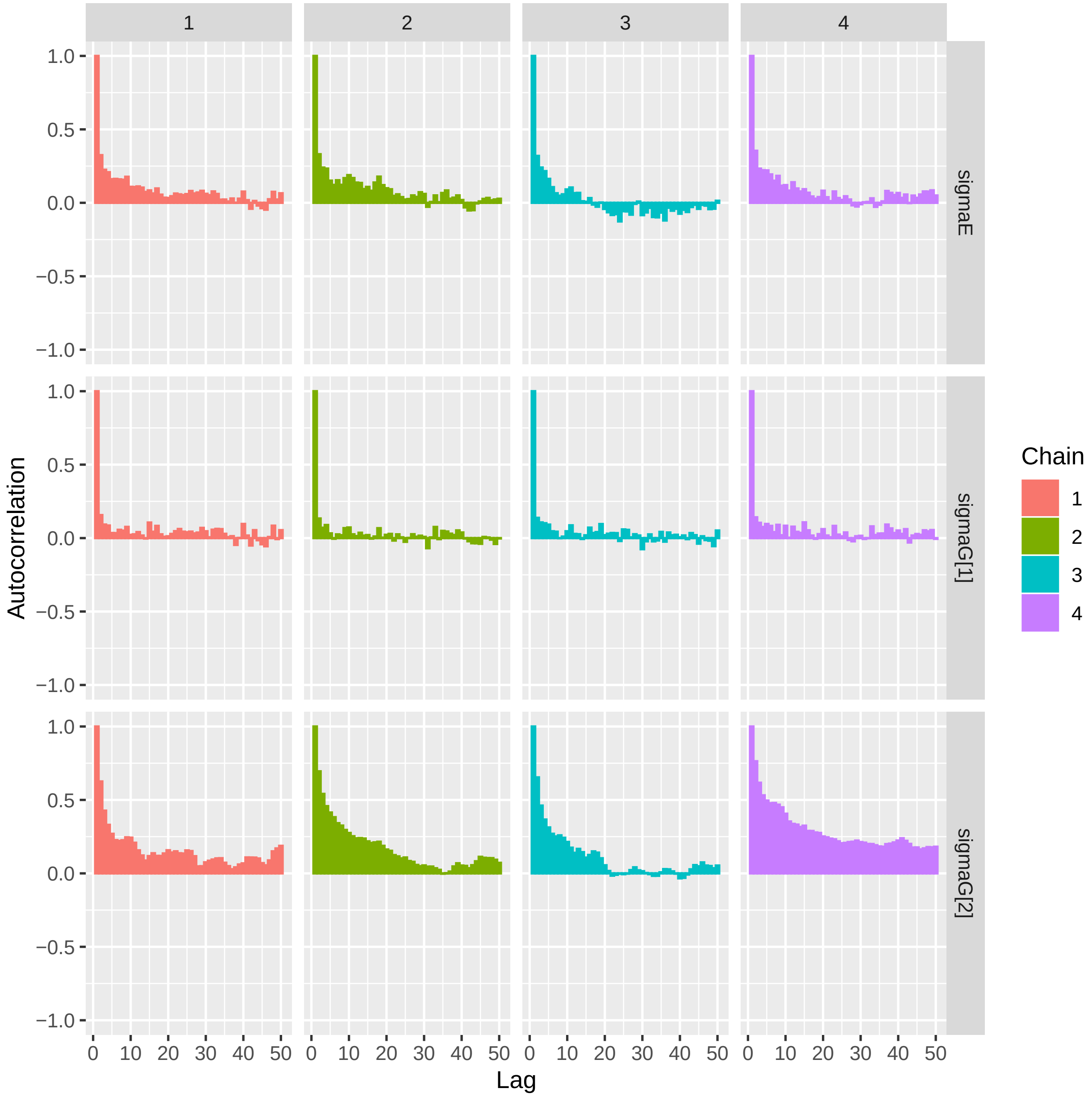

Supplementary Figure 11. Autocorrelation plot for the hyper-parameters of the smoking model in the Generation Scotland dataset.Four chains for BMI were run for 20000 samples with a burn-in of 10000 samples and thinning of 10 samples. Row panels give the autocorrelation for the error variance $sigmaE(\sigma_\epsilon^2)$, the variance attributable to SNP marker effects $sigmaG[2](\sigma_G^2)$, and the phenotypic variance associated with methylation probes $sigmaG[1](\sigma_\phi^2)$.

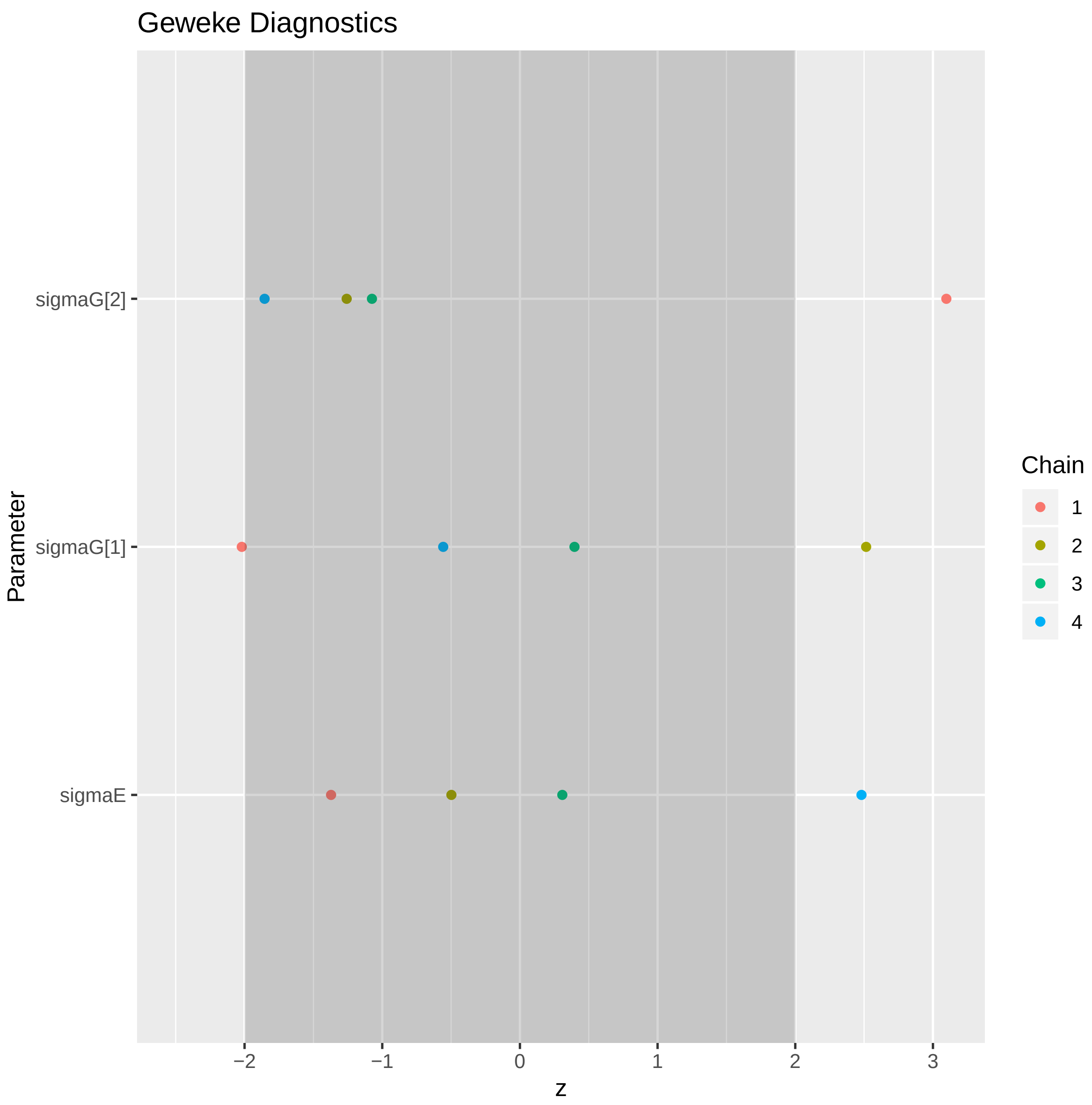

Supplementary Figure 12. Geweke z-score diagnostic for the hyper-parameters of the smoking model in the Generation Scotland dataset. Results for the four chains for the estimates of the the error variance $sigmaE(\sigma^2_\epsilon)$, the variance attributable to SNP marker effects $sigmaG[2](\sigma^2_G)$, and the phenotypic variance associated with methylation probes $sigmaG[1](\sigma^2_\phi)$, were compared using the Geweke z-score diagnostic with the acceptance interval(-2,2) highlighted in dark gray. Most chains reached the same parameter estimates, although there is some evidence of chain-level variance, meaning some effect of the starting values.

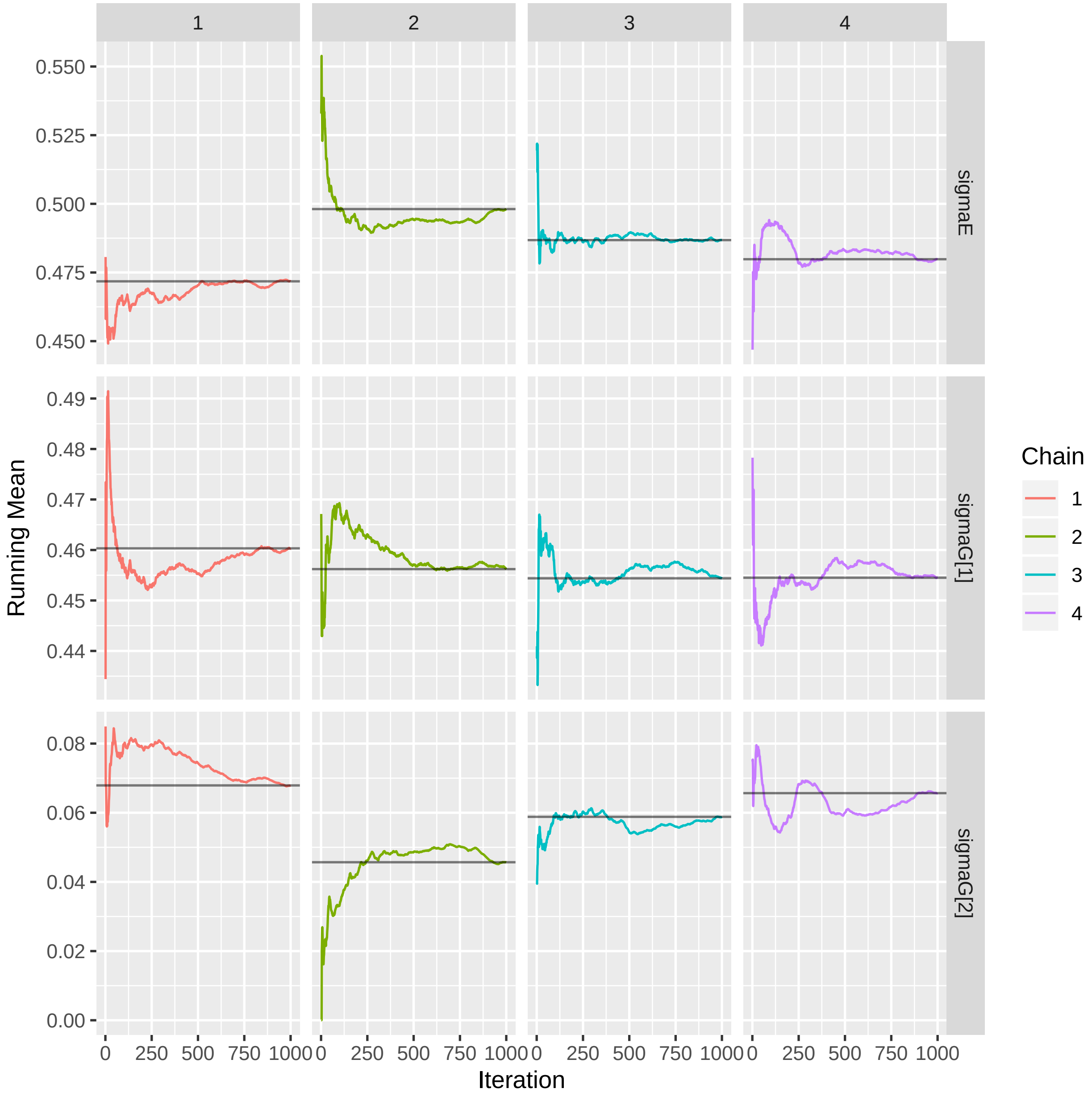

Supplementary Figure 13. Running means plot for the hyper-parameters of the smoking model in the Generation Scotland dataset. The mean of the chain at each sample is compared against the chains mean(horizontal line) for the estimates of tthe error variance $sigmaE(\sigma_\epsilon^2)$, the variance attributable to SNP marker effects $sigmaG[2](\sigma_G^2)$, and the phenotypic variance associated with methylation probes $sigmaG[1](\sigma_\phi^2)$. Given this, and the evidence of the among chain variance we combined the chains were thinned further to achieve 100 samples per chain.

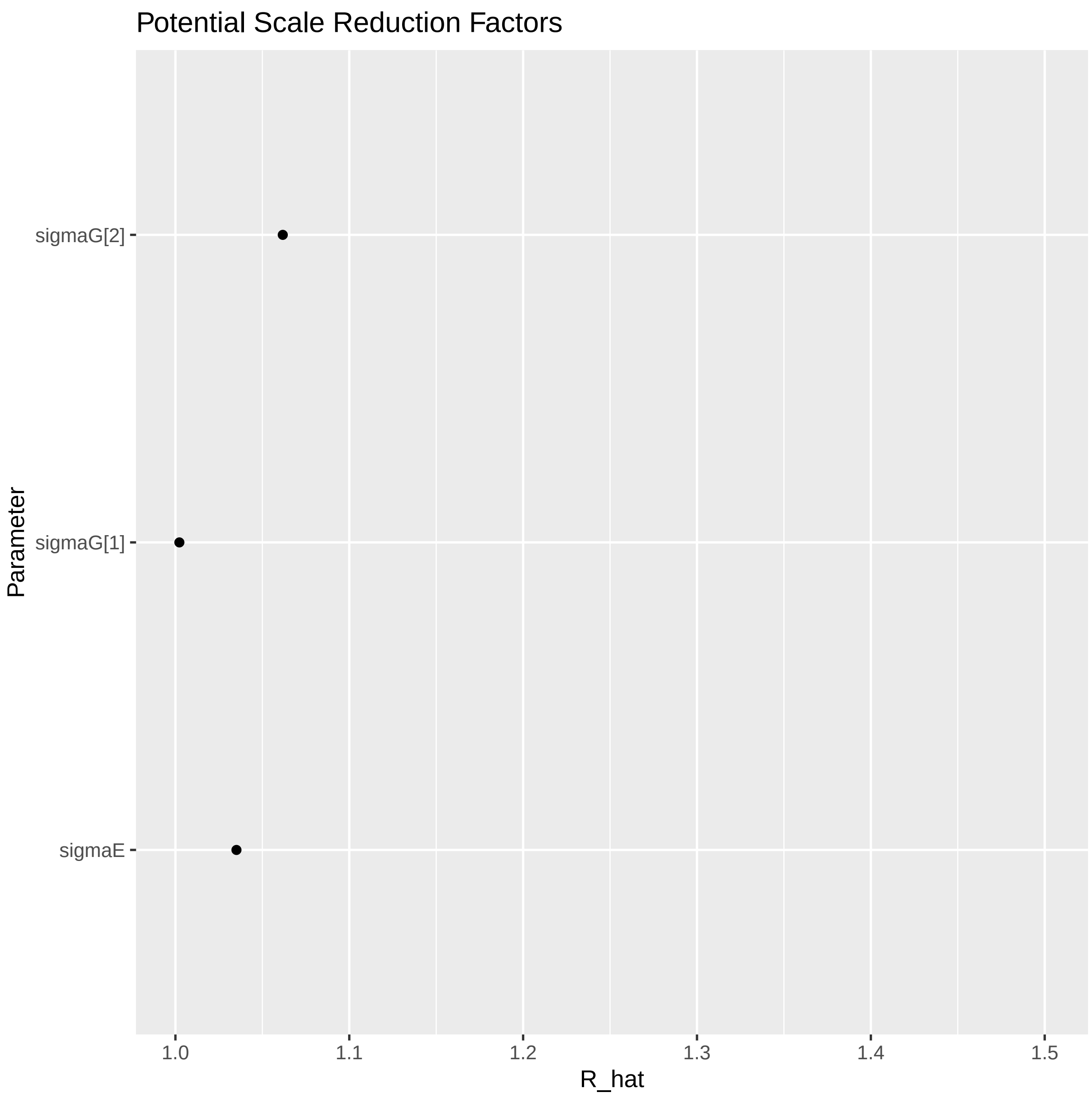

Supplementary Figure 14. Gelman R_{hat} diagnostic for the hyper-parameters of the smoking trait model in the Generation Scotland dataset. Results for the four chains for the estimates of the the error variance $sigmaE(\sigma_\epsilon^2)$, the variance attributable to SNP marker effects $sigmaG[2](\sigma_G^2)$, and the phenotypic variance associated with methylation probes $sigmaG[1](\sigma_\phi^2)$, were compared using the R_{hat} diagnostic, values lower than 1.1 are suggestive of convergence between chains.

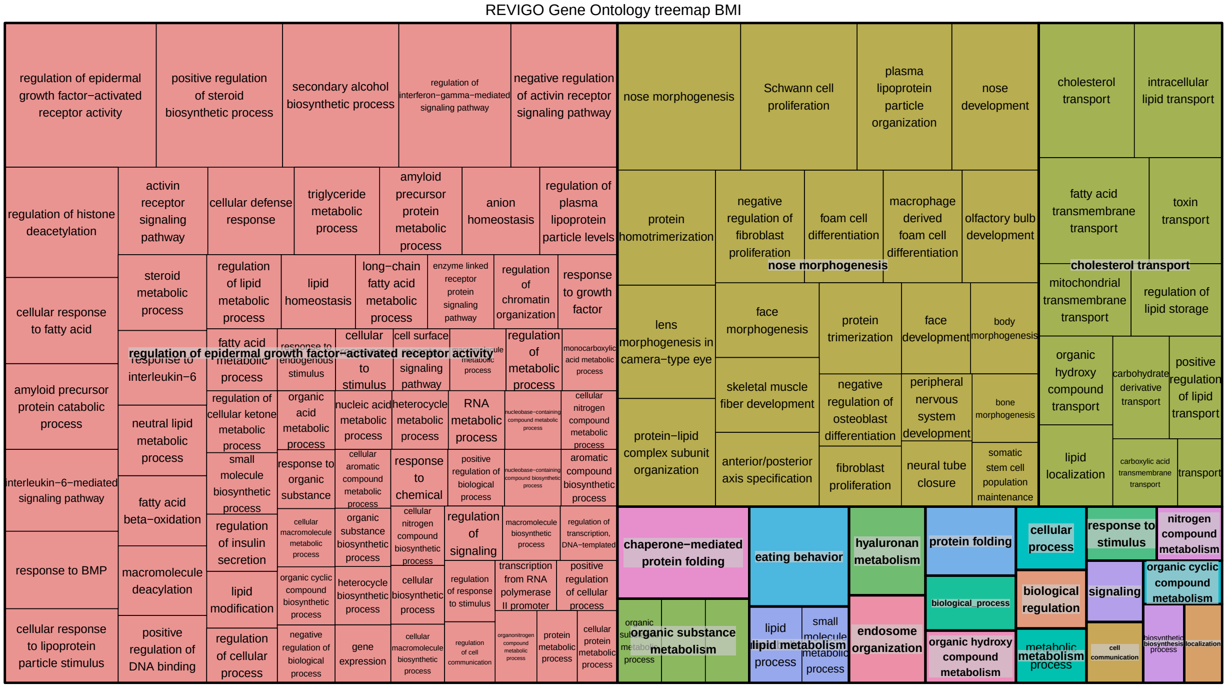

Supplementary Figure 15. Treemap of the top GO enriched terms for BMI(enrichment>2.0 and PIP>95%). Plot generated with REVIGO

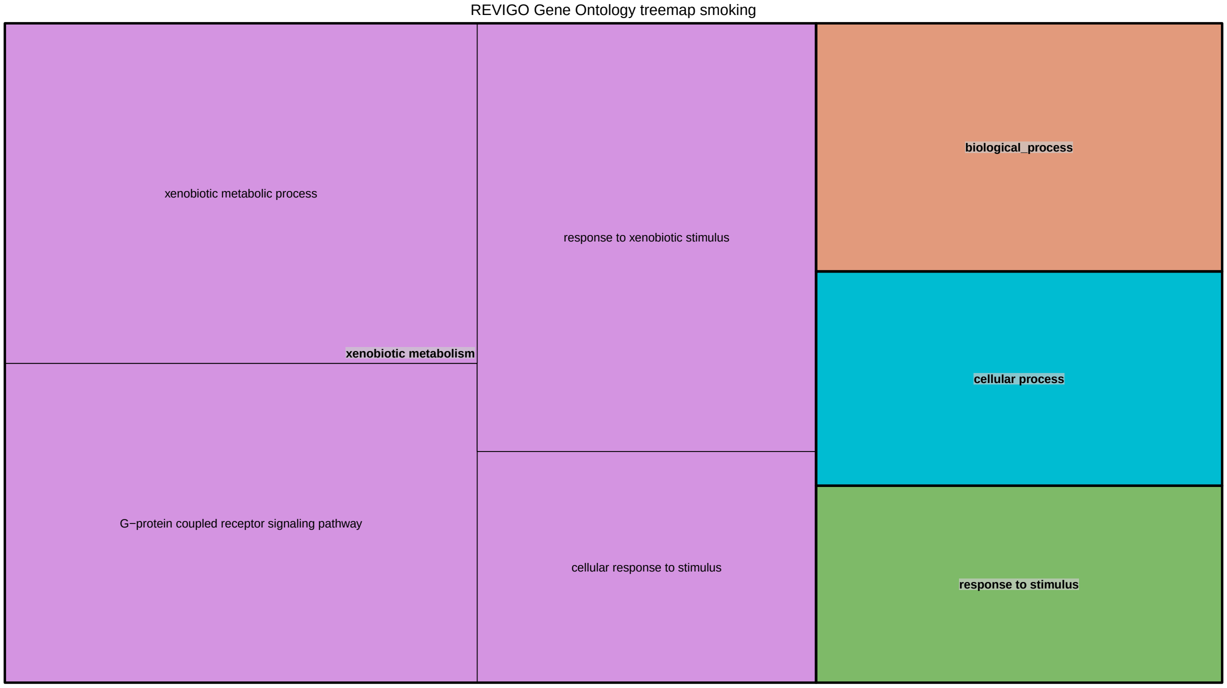

Supplementary Figure 16. Treemap of the top GO enriched terms for smoking(enrichment>2.0 and PIP>95%). Plot generated with REVIGO

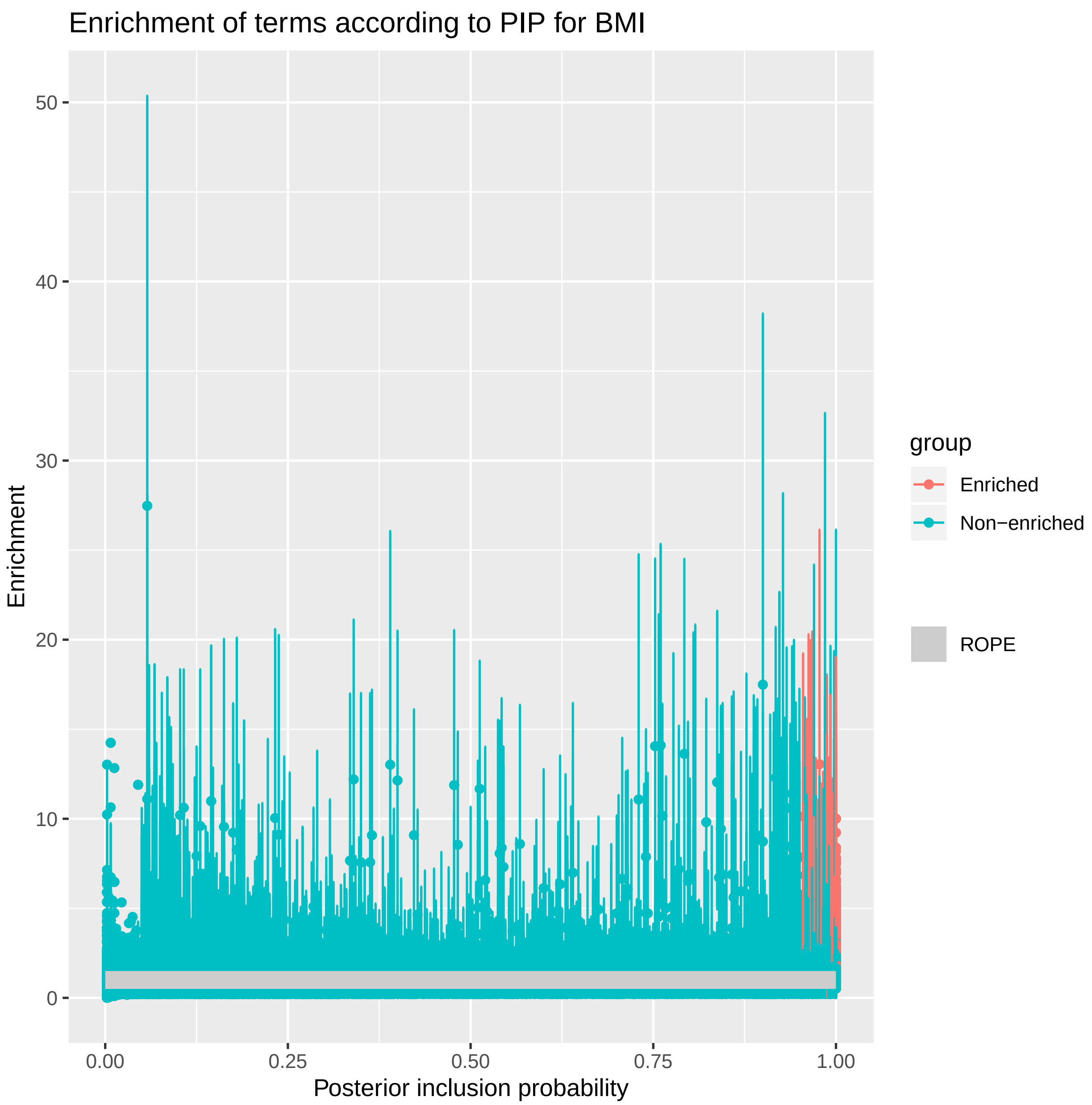

Supplementary Figure 17. Enrichment of GO terms for BMI, plotted are the mean terms enrichment and the 95% highest posterior density intervals against the posterior inclusion probability of the term, on gray the rope area around enrichment 1.0(0.5-1.5).

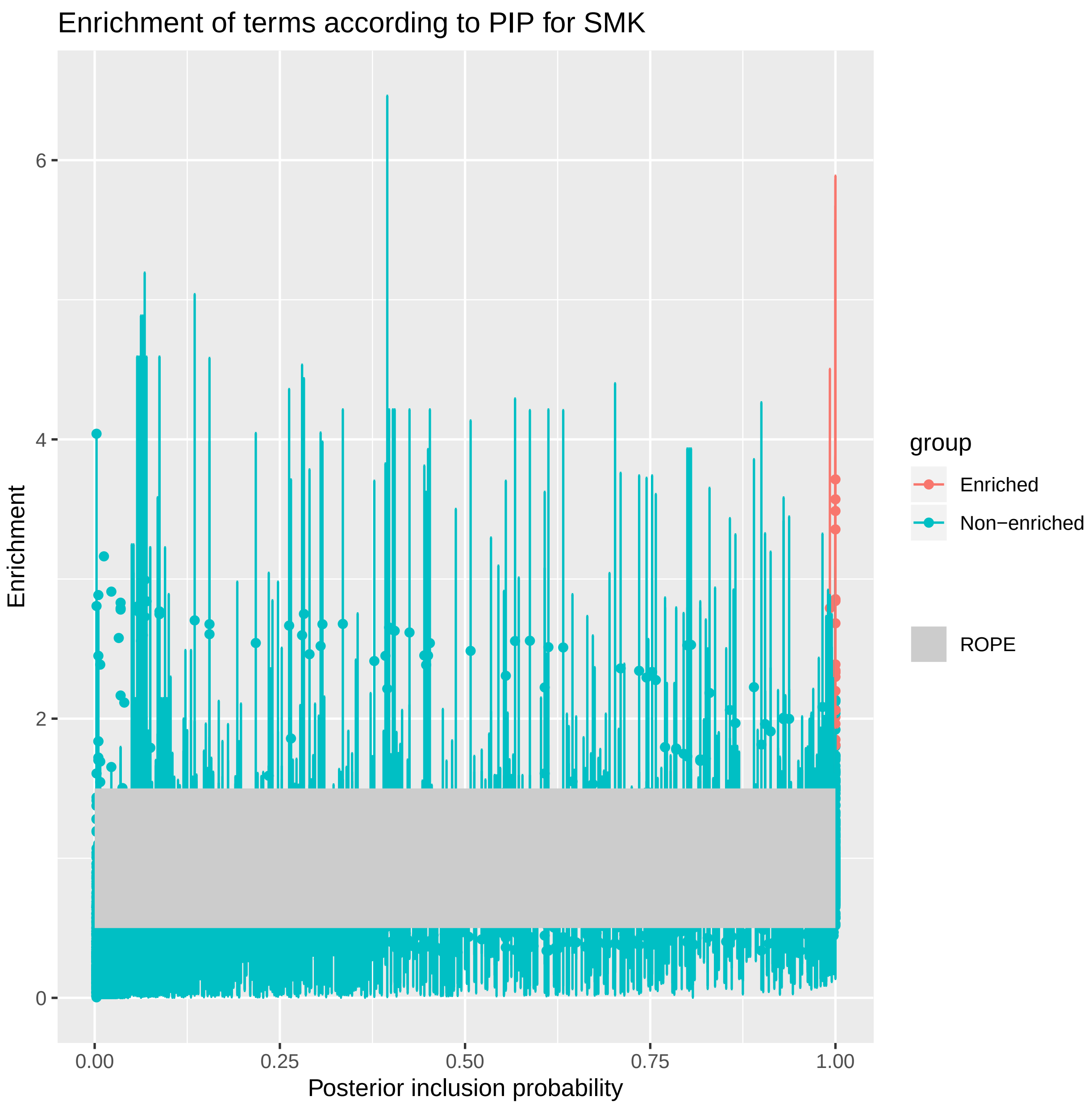

Supplementary Figure 18. Enrichment of GO terms for smoking, plotted are the mean terms enrichment and the 95% highest posterior density intervals against the posterior inclusion probability of the term, on gray the rope area around enrichment 1.0(0.5-1.5).

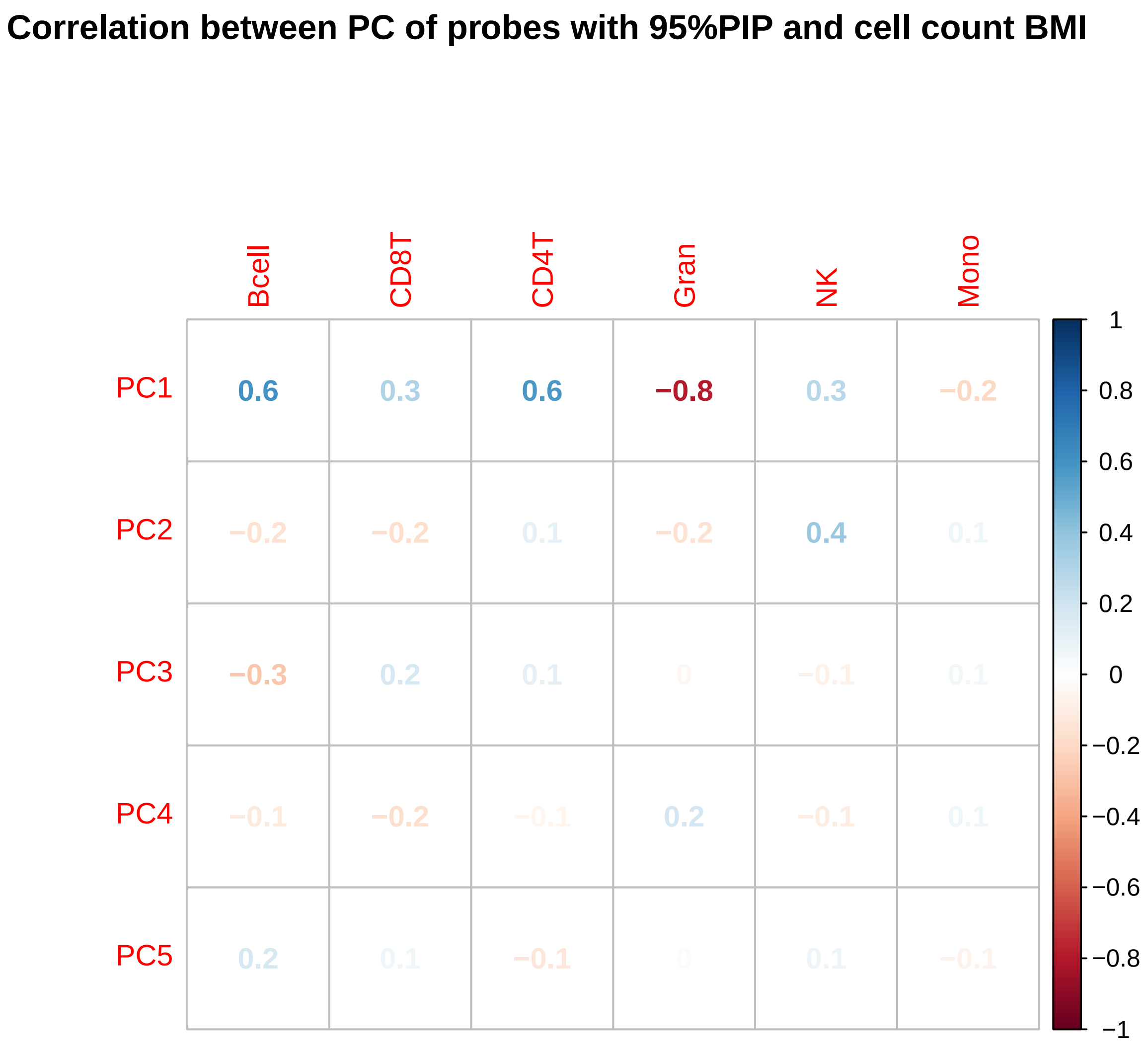

Supplementary Figure 19. Correlation between first 5 PCS of probes with 95% PIP for BMI and the cell counts.

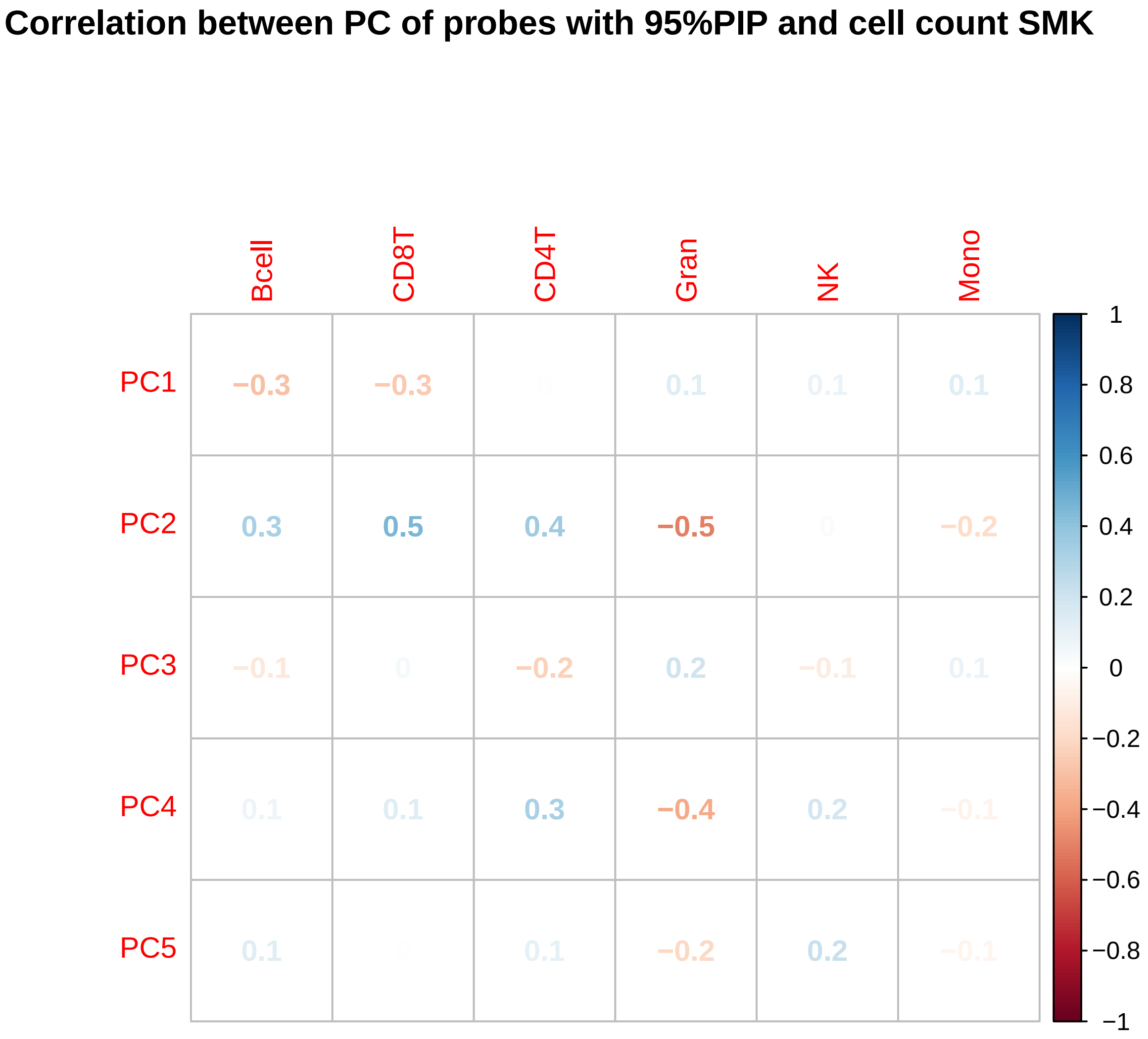

Supplementary Figure 20. Correlation between first 5 PCS of probes with 95% PIP for smoking and the cell counts.

Tables

	Min.	1st Qu.	Median	Mean	3rd Qu.	Max.	CI(l)	CI(h)
Markers in model	36070.00	65182.25	85209.50	79681.51	92599.75	103253.00	49688.00	100246.00
cpg probes in model	1795.00	3694.00	3973.50	3986.71	4362.00	5346.00	2960.00	5028.00
SNP in model	32196.00	61274.50	81280.00	75682.71	88594.50	98437.00	45383.00	95985.00
SNP in mixture 1	25423.00	57403.75	80236.50	73502.01	87889.75	98203.00	39565.00	95343.00
SNP in mixture 2	14.00	664.00	1279.50	2123.22	3675.25	6969.00	14.00	5526.00
SNP in mixture 3	0.00	22.00	44.00	57.49	79.00	275.00	0.00	154.00
cpg probes in mixture 1	975.00	3089.25	3443.00	3458.25	3889.50	4943.00	2320.00	4588.00
cpg probes in mixture 2	280.00	456.00	510.00	509.33	560.25	802.00	348.00	663.00
cpg probes in mixture 3	1.00	13.00	18.00	19.12	25.00	56.00	4.00	37.00
VE(%) SNP	8.86	14.24	15.79	15.83	17.31	22.64	11.24	20.58
VE(%) SNP in mixture 1	2.65	8.81	12.05	11.65	14.09	20.42	5.47	18.32
VE(%) SNP in mixture 2	0.01	1.02	2.05	3.36	5.73	11.29	0.12	8.92
VE(%) SNP in mixture 3	0.00	0.36	0.69	0.81	1.14	2.84	0.00	2.01
VE(%) cpg	69.23	74.47	75.86	75.70	77.12	81.96	71.73	79.31
VE(%) cpg in mixture 1	7.03	23.10	26.08	26.24	29.74	38.73	17.43	37.31
VE(%) cpg in mixture 2	22.61	35.94	39.49	39.59	43.57	58.71	28.37	49.47
VE(%) cpg in mixture 3	0.00	7.90	9.76	10.08	12.52	21.76	3.79	17.93
VE(%) by probes with 95% IP	6.86	8.99	9.69	9.70	10.33	13.24	7.55	11.93
(%) genome mapping	3.05	6.01	6.43	6.43	7.00	8.46	4.91	7.95

Supplementary Table 1. BMI posterior summary

	Min.	1st Qu.	Median	Mean	3rd Qu.	Max.	CI(l)	CI(h)
Markers in model	558.00	2255.00	4439.50	4495.16	6485.25	9607.00	960.00	8503.00
cpg probes in model	81.00	106.00	115.00	115.35	124.00	162.00	89.00	139.00
SNP in model	428.00	2120.25	4328.50	4379.61	6360.50	9502.00	815.00	8363.00
SNP in mixture 1	65.00	1520.00	3994.50	4007.96	6205.25	9493.00	215.00	8309.00
SNP in mixture 2	0.00	139.50	307.50	349.19	517.75	1021.00	0.00	778.00
SNP in mixture 3	0.00	10.00	18.00	22.46	29.00	120.00	0.00	56.00
cpg probes in mixture 1	79.00	102.00	110.00	111.34	120.00	161.00	87.00	140.00
cpg probes in mixture 2	0.00	1.00	3.00	3.42	5.00	15.00	0.00	9.00
cpg probes in mixture 3	0.00	0.00	0.00	0.58	1.00	5.00	0.00	2.00
VE(%) SNP	0.00	4.03	5.58	5.96	7.68	18.67	0.00	11.04
VE(%) SNP in mixture 1	0.00	0.70	1.93	2.73	3.76	17.23	0.00	7.90
VE(%) SNP in mixture 2	0.00	0.56	1.50	2.06	3.00	10.70	0.00	5.69
VE(%) SNP in mixture 3	0.00	0.80	1.13	1.15	1.48	3.12	0.00	2.16
VE(%) cpg	35.21	43.12	45.66	45.61	48.15	57.18	37.36	51.96
VE(%) cpg in mixture 1	19.65	30.73	34.77	35.15	39.23	51.99	24.00	46.79
VE(%) cpg in mixture 2	0.00	0.00	5.38	5.58	9.66	18.63	0.00	14.27
VE(%) cpg in mixture 3	0.00	0.00	0.00	0.72	0.00	12.73	0.00	7.53
VE(%) by probes with 95% IP	21.01	25.22	26.72	26.78	28.21	33.75	22.36	30.90
(%) genome mapping	0.13	0.19	0.20	0.20	0.22	0.31	0.15	0.24

Supplementary Table 2. Smoking posterior summary

	0<5%	5%<50%	50%<95%	95%>
BMI	364274	563283	50	15
smoking	555706	167	17	17

Supplementary Table 3. Number of probes with posterior inclusion probabilities in ranges

	ONTOLOGY	TERM	N	DE	P.DE	FDR
GO:0000814	CC	ESCRT II complex	3	1	0	1
GO:0009617	BP	response to bacterium	89	2	0	1
GO:0009720	BP	detection of hormone stimulus	1	1	0	1
GO:0034041	MF	sterol-transporting ATPase activity	1	1	0	1
GO:0071403	BP	cellular response to high density lipoprotein particle stimulus	2	1	0	1
GO:0060335	BP	positive regulation of interferon-gamma-mediated signaling pathway	6	1	0	1
GO:0004095	MF	carnitine O-palmitoyltransferase activity	2	1	0	1
GO:0046811	MF	histone deacetylase inhibitor activity	2	1	0	1
GO:0010872	BP	regulation of cholesterol esterification	4	1	0	1
GO:1990698	MF	palmitoleoyltransferase activity	2	1	0	1
GO:0045345	BP	positive regulation of MHC class I biosynthetic process	5	1	0	1
GO:0070062	CC	extracellular exosome	2162	5	0	1
GO:0031064	BP	negative regulation of histone deacetylation	5	1	0	1
GO:0034437	MF	glycoprotein transporter activity	3	1	0	1
GO:0042987	BP	amyloid precursor protein catabolic process	8	1	0	1
GO:0019534	MF	toxin transmembrane transporter activity	5	1	0	1
GO:0046983	MF	protein dimerization activity	158	2	0	1
GO:0009437	BP	carnitine metabolic process	4	1	0	1
GO:0034436	BP	glycoprotein transport	4	1	0	1
GO:0032367	BP	intracellular cholesterol transport	10	1	0	1

Supplementary Table 6. Top 20 GO enrichment terms for the probes with 95%IP in BMI, column DE number of genes that are differentially methylated and P.DE. means p-valuep-value for over-representation of the gene set for the term, FDR means false discovery rate.

	ONTOLOGY	TERM	N	DE	P.DE	FDR
GO:0021987	BP	cerebral cortex development	62	2	0	1
GO:0007626	BP	locomotory behavior	83	2	0	1
GO:0090251	BP	protein localization involved in establishment of planar polarity	1	1	0	1
GO:0005886	CC	plasma membrane	4271	7	0	1
GO:0015057	MF	thrombin-activated receptor activity	5	1	0	1
GO:0071109	BP	superior temporal gyrus development	2	1	0	1
GO:0042249	BP	establishment of planar polarity of embryonic epithelium	3	1	0	1
GO:0021761	BP	limbic system development	2	1	0	1
GO:0060488	BP	orthogonal dichotomous subdivision of terminal units involved in lung branching morphogenesis	2	1	0	1
GO:0060489	BP	planar dichotomous subdivision of terminal units involved in lung branching morphogenesis	2	1	0	1
GO:0060490	BP	lateral sprouting involved in lung morphogenesis	2	1	0	1
GO:0046983	MF	protein dimerization activity	158	2	0	1
GO:0045163	BP	clustering of voltage-gated potassium channels	3	1	0	1
GO:0048133	BP	male germ-line stem cell asymmetric division	4	1	0	1
GO:0072678	BP	T cell migration	8	1	0	1
GO:0060155	BP	platelet dense granule organization	8	1	0	1
GO:0060762	BP	regulation of branching involved in mammary gland duct morphogenesis	5	1	0	1
GO:0048105	BP	establishment of body hair planar orientation	4	1	0	1
GO:0003924	MF	GTPase activity	224	2	0	1
GO:0071340	BP	skeletal muscle acetylcholine-gated channel clustering	8	1	0	1

Supplementary Table 7. Top 20 GO enrichment terms for the probes with 95%IP in smoking, column DE number of genes that are differentially methylated and P.DE. means p-valuep-value for over-representation of the gene set for the term, FDR means false discovery rate.

	cpg	PIP	symbol	group	alias	entrezid
8079	cg00574958	1.00	CPT1A	5'UTR	CPT1A	1374
46935	cg03546163	1.00	FKBP5	5'UTR	FKBP5	2289
77359	cg05991220	1.00	ITIH1	TSS200	ITIH1	3697
83016	cg06500161	1.00	ABCG1	Body	ABCG1	9619
112034	cg08857797	1.00	VPS25	Body	VPS25	84313
188228	cg15564619	0.99	SKI	Body	SKI	6497
200687	cg16663980	0.99	MNDA	5'UTR	MNDA	4332
99997	cg07839457	0.98	NLRC5	TSS1500	NLRC5	84166
16470	cg01199327	0.97	CD82	Body	CD82	3732
294554	cg25648203	0.96	AHRR	Body	AHRR	57491
308863	cg26950531	0.94	DPF1	Body	DPF1	8193
216721	cg18181703	0.94	SOCS3	Body	SOCS3	9021
79575	cg06192883	0.93	MYO5C	Body	MYO5C	55930
269672	cg23320499	0.93	DUSP16	Body	DUSP16	80824
23882	cg01787285	0.92	SKI	Body	SKI	6497
300766	cg26253134	0.92	TGFA	Body	TGFA	7039
136457	cg11024682	0.91	SREBF1	Body	SREBF1	6720
209587	cg17501210	0.91	RPS6KA2	Body	RPS6KA2	6196
227392	cg19202384	0.91	PYCR1	Body	PYCR1	5831
36270	cg02714303	0.89	LMO7	TSS200	LMO7	4008
250969	cg21507987	0.88	AMPD3	3'UTR	AMPD3	272
213567	cg17901584	0.88	DHCR24	TSS1500	DHCR24	1718
204757	cg17058475	0.83	CPT1A	5'UTR	CPT1A	1374
53516	cg04065210	0.80	DPY19L1	Body	DPY19L1	23333
99120	cg07769588	0.79	ATG4D	Body	ATG4D	84971
172490	cg14195115	0.78	SHB	Body	SHB	6461
309115	cg26971585	0.77	PRF1	Body	PRF1	5551
165566	cg13680388	0.76	GNAS	Body	GNAS	2778
213086	cg17858328	0.74	GFI1	TSS1500	GFI1	2672
48163	cg03636183	0.73	F2RL3	Body	F2RL3	9002
207198	cg17287155	0.72	AHRR	Body	AHRR	57491
78458	cg06092244	0.70	STON1-GTF2A1L	TSS200	STON1-GTF2A1L	286749
297101	cg25921813	0.70	C3orf21	Body	XXYLT1	152002
169533	cg13981001	0.69	RPL12	Body	RPL12	6136
169534	cg13981001	0.69	SNORA65	TSS1500	SNORA65	26783
209327	cg17478979	0.67	ZC3H12D	Body	ZC3H12D	340152
270400	cg23384027	0.66	NFE2	Body	NFE2	4778
24639	cg01839993	0.62	DDIT4	Body	DDIT4	54541
211170	cg17669033	0.62	IL1RN	1stExon	IL1RN	3557
97304	cg07618085	0.61	TBC1D16	Body	TBC1D16	125058
8423	cg00594099	0.60	AHDC1	5'UTR	AHDC1	27245
246686	cg21085022	0.57	C6orf106	Body	ILRUN	64771
57463	cg04367503	0.56	NFIX	Body	NFIX	4784
162799	cg13478588	0.54	BAT2	Body	PRRC2A	7916
2152	cg00144022	0.54	BCL7A	Body	BCL7A	605
72682	cg05603985	0.51	SKI	1stExon	SKI	6497
227461	cg19211853	0.51	RBM26	Body	RBM26	64062

Supplementary Table 4. Probes with IP $>$ 50% for BMI

	cpg	PIP	symbol	group	alias	entrezid
48163	cg03636183	1.00	F2RL3	Body	F2RL3	9002
72334	cg05575921	1.00	AHRR	Body	AHRR	57491
138402	cg11207515	1.00	CNTNAP2	Body	CNTNAP2	26047
184306	cg15159987	1.00	CPAMD8	3'UTR	CPAMD8	27151
269367	cg23288337	1.00	ECEL1P2	Body	ECEL1P2	347694
289814	cg25189904	1.00	GNG12	TSS1500	GNG12	55970
305915	cg26703534	1.00	AHRR	Body	AHRR	57491
102314	cg08038054	1.00	GNG11	TSS1500	GNG11	2791
294554	cg25648203	1.00	AHRR	Body	AHRR	57491
12189	cg00884093	0.99	CELSR1	1stExon	CELSR1	9620
57865	cg04400533	0.99	ETV5	Body	ETV5	2119
234540	cg19859270	0.98	GPR15	1stExon	GPR15	2838
307626	cg26850624	0.93	AHRR	Body	AHRR	57491
161437	cg13378934	0.91	MEIS3	Body	MEIS3	56917
80092	cg06235438	0.89	ITGAL	Body	ITGAL	3683
241325	cg20543544	0.83	ZMIZ1	Body	ZMIZ1	57178
48627	cg03669698	0.83	GAS2L1	3'UTR	GAS2L1	10634
231264	cg19572487	0.80	RARA	5'UTR	RARA	5914
168980	cg13937905	0.74	RARG	Body	RARG	5916
245751	cg20988565	0.74	ZFPM2	Body	ZFPM2	23414
258032	cg22207272	0.70	CADM1	TSS1500	CADM1	23705
270071	cg23351584	0.69	PRSS23	5'UTR	PRSS23	11098
7049	cg00501876	0.61	CSRNP1	5'UTR	CSRNP1	64651
234752	cg19877205	0.57	PFDN4	1stExon	PFDN4	5203
222903	cg18754985	0.56	CLDND1	Body	CLDND1	56650
21871	cg01620785	0.54	C2orf28	Body	ATRAID	51374
21872	cg01620785	0.54	SLC5A6	TSS1500	SLC5A6	8884

Supplementary Table 5. Probes with IP $>$ 50% for smoking

	go_id	Min.	1st Qu.	Median	Mean	3rd Qu.	Max.	CI(l)	CI(h)	ROPE	prob
1	GO:0007176	1.26	8.60	11.55	13.05	16.51	45.07	2.61	26.13	0.00	0.98
2	GO:0010893	2.69	7.70	9.94	10.90	13.01	30.60	3.75	20.45	0.00	0.97
3	GO:0043585	3.52	7.74	10.14	10.83	13.15	27.36	4.65	20.30	0.00	0.96
4	GO:0010624	3.54	7.57	9.67	10.56	12.55	26.84	4.19	19.96	0.00	0.96
5	GO:0010626	3.54	7.57	9.67	10.56	12.55	26.84	4.19	19.96	0.00	0.96
6	GO:0014010	3.54	7.33	9.21	10.23	12.28	26.84	3.54	18.54	0.00	0.96
7	GO:0016859	2.47	7.36	9.40	10.13	12.12	29.12	4.24	19.23	0.00	0.95
8	GO:1902653	2.73	7.30	9.32	10.01	12.18	32.10	3.04	19.03	0.00	1.00
9	GO:0006695	2.73	7.28	9.30	10.00	12.18	32.10	3.04	19.03	0.00	1.00
10	GO:0045940	2.42	6.86	8.94	9.75	11.66	30.60	3.30	18.00	0.00	0.99
11	GO:0060330	1.71	6.42	8.68	9.68	11.91	34.99	2.69	18.40	0.00	0.96
12	GO:0060334	1.71	6.40	8.66	9.66	11.91	34.99	2.69	18.40	0.00	0.96
13	GO:0016126	2.36	6.60	8.63	9.23	11.36	26.75	2.81	17.22	0.00	1.00
14	GO:0032926	3.03	6.48	8.24	9.19	10.93	33.74	3.03	17.28	0.00	0.99
15	GO:1902932	2.27	6.30	8.01	9.03	10.97	26.30	2.90	16.92	0.00	0.99
16	GO:0030514	3.49	6.18	7.74	8.39	9.86	23.54	3.63	14.08	0.00	1.00
17	GO:0071827	2.02	5.76	7.68	8.33	9.98	34.12	2.94	16.94	0.00	0.98
18	GO:0042059	0.88	5.47	7.28	8.18	9.98	27.91	2.11	15.99	0.00	1.00
19	GO:0045540	2.36	5.49	7.04	7.81	9.23	22.89	2.36	14.78	0.00	1.00
20	GO:0106118	2.36	5.49	7.04	7.81	9.23	22.89	2.36	14.78	0.00	1.00
21	GO:0030301	2.27	5.30	7.21	7.70	9.33	20.69	2.89	13.61	0.00	1.00

Supplementary Table 8. Top enriched terms for BMI, ranked by the highest posterior mean enrichment.

	go_id	Min.	1st Qu.	Median	Mean	3rd Qu.	Max.	CI(l)	CI(h)	ROPE	prob
1	GO:0006805	1.53	2.95	3.60	3.71	4.28	7.53	2.11	5.86	0.00	1.00
2	GO:0071466	1.53	2.84	3.40	3.57	4.14	7.53	1.84	5.67	0.00	1.00
3	GO:0007186	1.42	2.68	3.37	3.49	4.17	7.24	1.97	5.89	0.00	1.00
4	GO:0009410	1.44	2.59	3.20	3.36	3.97	7.13	1.84	5.58	0.00	1.00
5	GO:0140110	1.44	2.51	2.81	2.86	3.19	4.69	1.94	3.89	0.01	1.00
6	GO:0003700	1.51	2.48	2.81	2.84	3.14	4.43	1.95	3.87	0.00	1.00
7	GO:0004930	1.19	2.22	2.72	2.79	3.27	5.39	1.62	4.50	0.02	0.99
8	GO:0000981	1.39	2.36	2.64	2.68	2.96	4.21	1.80	3.63	0.01	1.00
9	GO:0003674	1.87	2.25	2.38	2.39	2.52	3.05	2.03	2.80	0.00	1.00
10	GO:0005654	1.38	2.03	2.30	2.34	2.62	3.99	1.55	3.21	0.01	1.00
11	GO:0008150	1.90	2.22	2.32	2.33	2.44	2.98	2.03	2.68	0.00	1.00
12	GO:0031981	1.37	2.00	2.27	2.30	2.54	3.68	1.55	3.07	0.01	1.00
13	GO:0044428	1.39	1.92	2.16	2.20	2.43	3.41	1.53	2.89	0.01	1.00
14	GO:0031974	1.30	1.82	2.04	2.06	2.26	3.30	1.41	2.72	0.04	1.00
15	GO:0043233	1.30	1.82	2.04	2.06	2.26	3.30	1.41	2.72	0.04	1.00
16	GO:0070013	1.30	1.82	2.04	2.06	2.26	3.30	1.41	2.72	0.04	1.00
17	GO:0009987	1.61	1.89	2.01	2.01	2.11	2.64	1.73	2.32	0.00	1.00
18	GO:0005575	1.62	1.87	1.95	1.96	2.06	2.33	1.69	2.26	0.00	1.00
19	GO:0050896	1.36	1.73	1.84	1.85	1.94	2.41	1.53	2.22	0.02	1.00
20	GO:0051716	1.31	1.69	1.81	1.81	1.92	2.35	1.43	2.16	0.04	1.00
21	GO:0044464	1.48	1.71	1.81	1.81	1.91	2.15	1.54	2.07	0.01	1.00
22	GO:0005623	1.48	1.71	1.80	1.81	1.91	2.15	1.54	2.07	0.01	1.00

Supplementary Table 9. Top enriched terms for smoking, ranked by the highest posterior mean enrichment.
